# Optimized Hesperidin-Loaded Lipid Nanoparticles with Tea Tree Oil for Enhanced Wound Healing: Formulation, Characterization, and Evaluation

**DOI:** 10.3390/ph18030290

**Published:** 2025-02-20

**Authors:** Borros Arneth, Rehab Abdelmonem, Mohamed A. El-Nabarawi, Mahmoud Hassan Teaima, Kareem Omar Rashwan, Mohamed A. Soliman, Inas Essam Ibrahim Al-Samadi

**Affiliations:** 1 Institute of Laboratory Medicine and Pathobiochemistry, Molecular Diagnostics, Philipps University Marburg, 35043 Marburg, Germany; 2 Institute of Laboratory Medicine and Pathobiochemistry, Molecular Diagnostics, Justus Liebig University Giessen, 35392 Giessen, Germany; 3 Department of Industrial Pharmacy, College of Pharmaceutical Sciences and Drug Manufacturing, Misr University for Science and Technology, Giza 12566, Egypt; rehab.abdelmonem@must.edu.eg (R.A.); mohamed.abdelgalil@must.edu.eg (M.A.S.); inas.samadi@must.edu.eg (I.E.I.A.-S.); 4 Department of Pharmaceutics and Industrial Pharmacy, Faculty of Pharmacy, Cairo University, Giza 11562, Egypt; mohamed.elnabarawi@pharma.cu.edu.eg (M.A.E.-N.);; 5 Department of Pharmaceutics and Industrial Pharmacy, Faculty of Pharmacy, October 6 University, Giza 12585, Egypt; kareemomar64@gmail.com

**Keywords:** wound healing, hesperidin, solid lipid nanoparticles, nanostructure liquid carriers, tea tree oil, anti-inflammatory, in vivo study, cytotoxicity, biomarkers

## Abstract

**Objectives:** This study aimed to develop hesperidin solid lipid nanoparticles (HESP-SLNs) to enhance their stability, solubility, and sustained release for wound healing; further enhancement was achieved through prepared nanostructured lipid carriers (HESP-NLCs) using Tea Tree Oil (TTO) to explore their synergistic efficacy. **Methods**: A factorial design of 2^4^ trials was established to evaluate the influence of lipid type (X1), lipid conc (%) (X2), surfactant type (X3), and sonication amplitude (%) (X4) of prepared HESP-SLNs on the particle size (nm) (Y1), polydispersibility index (Y2), zeta potential (Y3), and encapsulation efficiency (%) (Y4). The optimized HESP-SLNs formula was selected utilizing Design Expert^®^ software version 13, which was additionally enhanced by preparing TTO-loaded HESP-NLCs. In vitro release, Raman spectroscopy, and transmission electron microscopy were carried out for both lipid nanoparticles. Cytotoxicity, in vivo wound-healing assessments, and skin irritancy tests were performed to evaluate the performance of TTO-incorporated HESP-NLCs compared to HESP-SLNs. **Results:** The optimized formula demonstrated PS (280 ± 1.35 nm), ZP (−39.4 ± 0.92 mV), PDI (0.239 ± 0.012), and EE% (88.2 ± 2.09%). NLCs enhanced Q6% release, (95.14%) vs. (79.69%), for SLNs and showed superior antimicrobial efficacy. Both lipid nanoparticles exhibited spherical morphology and compatibility between HESP and excipients. NLCs achieved the highest wound closure percentage, supported by histological analysis and inflammatory biomarker outcomes. Cytotoxicity evaluation showed 87% cell viability compared to untreated HSF cells, and the skin irritancy test confirmed the safety of NLCs. **Conclusions:** TTO-loaded HESP-NLCs are promising candidates exhibiting superior wound-healing capabilities, making them a potential therapeutic option for cutaneous wound management.

## 1. Introduction

Skin is recognized as the largest organ in the human body, consisting of the dermis, epidermis, and ancillary structures [1]. When the skin is exposed to continuous destruction from external insults or is weakened owing to immune system dysregulation, it can form wounds [2]. A wound disrupts biological tissue, such as mucous membranes, skin, and different organs in the human body [3]. It determines severe morbidity and even mortality, especially in elderly persons; early intervention in wound management can reduce the risk of wound complications [4,5], which increases healthcare expenditures [6]. The predicted cost is estimated to be USD 18.7 billion by 2027, with a substantial portion of the healthcare expenses allocated to wound management [7]. In light of this, many efforts have been made to enhance the effectiveness of highly stable, inexpensive, and minimal side-effect treatments that promote tissue regeneration and reduce inflammation, as well as scar formation [8,9]. Therefore, synthetic compounds, including antibiotics, chlorhexidine, and silver, have been used to prevent wound infection, in addition to the natural products from various plant species, including honey, propolis, aloe vera, tea tree oil, and flavonoids, which were successfully studied in the management of cutaneous wounds, owing to their antimicrobial and anti-inflammatory efficacy [10,11].

Hesperidin (HESP) C_28_H_34_O_15_ is a kind of flavanone glycoside predominantly found in citrus fruits. It has been given remarkable attention owing to its several pharmacological characteristics, such as antimicrobial, antioxidant, and anti-inflammatory action [12,13,14]. Furthermore, it reduces scar formation and fibrosis after wound healing in experimental animals [15]. Despite hesperidin’s promising pharmacological actions, its poor aqueous solubility, low bioavailability, and susceptibility to environmental influences such as pH, temperature, and light limit its therapeutic effectiveness [16]. Thus, it is critical to establish a novel therapeutic approach to overcoming these limitations and maximizing its wound-healing activity. Subsequently, advanced drug delivery systems have been explored.

Nanotechnology is emerging as a promising field for discovering new natural-based wound-healing therapies because of nanoparticles’ unique properties [17]. It has been an area of attention for developing promising entities that can overcome many obstacles of conventional natural products, such as solubility, stability, and toxicity, providing a targeted delivery [18]. Lipid nanoparticles are favored for cutaneous distribution because of their safe interactions and structural resemblance to the stratum corneum [19].

Solid lipid nanoparticles SLNs are solid core carriers that possess the capability to encapsulate both hydrophobic and hydrophilic drugs [20], which offer further properties such as controlled drug release and enhancing the solubilization of the active compounds [21,22]. On the other hand, nanostructured lipid carriers represent a modified generation of SLNs characterized by the encapsulation of an oily phase, composed of liquid lipids, within a solid matrix, facilitating the formation of a structured network that promotes drug solubility, non-toxicity, biocompatibility, and biodegradability, confirming the stability of the encapsulated compounds [23]. Furthermore, NLCs have effectively encapsulated liquid oils such as essential oils (Lavandula, Mentha, Rosmarinus, tea tree oil) [24,25].

A type of essential oil known as tea tree oil (TTO) is obtained from *Melaleuca alternifolia* foliage. TTO is a complex blend of lipid molecules, terpenes, and volatile oils. The predominant constituents of TTO comprise cycloolefins, in addition to enolic substances, such as terpinene-4-ol, c-terpinene, terpinene, 1,8-cineole, as well as a-terpineol [26]. It exhibits an antimicrobial, anti-inflammatory, anticarcinogenic, anti-neoplastic, and immunomodulatory action [27,28]. Nevertheless, TTO irritability, allergic responses, volatility, and instability when exposed to light or oxygen limit its use in pharmaceutical products. The incorporation of TTO in the NLCs delivers an effective method for improving physical stability [29].

Numerous previous studies have independently investigated the effects of hesperidin and tea tree oil (TTO) on wound healing. Hesperidin exhibits anti-inflammatory, antioxidant, and collagen-promoting properties that facilitate tissue repair, while TTO possesses antimicrobial activity that prevents infection and promotes tissue regeneration. When combined, these compounds synergistically accelerate tissue repair, mitigate inflammation, and enhance the overall healing process, suggesting their potential as a promising therapeutic strategy for wound management [30,31,32].

Consequently, this study aimed to develop, evaluate, and optimize hesperidin-loaded solid lipid nanoparticles (HESP-SLNs) using Design Expert^®^ software (version 13). To the best of our knowledge, no existing research has investigated the potential enhancement of wound healing through the combined therapeutic benefits of hesperidin and tea tree oil (TTO). Therefore, in the present study, TTO-loaded hesperidin nanostructured lipid carriers (HESP-NLCs) were formulated and characterized. Furthermore, this study highlights the potential of TTO-loaded HESP-NLCs as a safe and effective wound treatment. A comprehensive in vivo evaluation of both SLNs and NLCs was conducted in a rat model to compare their therapeutic efficacy and overall impact on wound healing.

## 2. Results and Discussion

### 2.1. Optimization of Factorial Design

The appropriate preliminary studies were conducted to identify the factors that could significantly affect the characteristics of the SLNs; therefore, the following variables were selected as independent variables. Lipid type (X1), lipid concentration (X2), surfactant type (X3), and sonication amplitude (X4) were chosen to be the independent variables for the design of the experiment, Table 1. Additionally, the value of adjusted R^2^ and predicted R^2^ of various responses were acceptable since the difference between each of them was fewer than 0.2. Moreover, adequate precision was noticed to be greater than the desired value (4) for Y1, Y2, Y3, and Y4. Therefore, this model could be suitable for the design space navigation, Table 2.

### 2.2. Investigation of HESP-SLNs

#### 2.2.1. Impact of Formulation Variables on PS

One of the most significant physical characteristics of SLNs is the PS, which can influence drug absorption and release patterns [33]. Based on the findings in Table 1, the particle size ranged from 252 ± 3.12 to 495 ± 1.70 nm. These outcomes agreed with previous research that determined SLNs had a diameter between 50 and 1000 nm [34].

**Figure 1:**
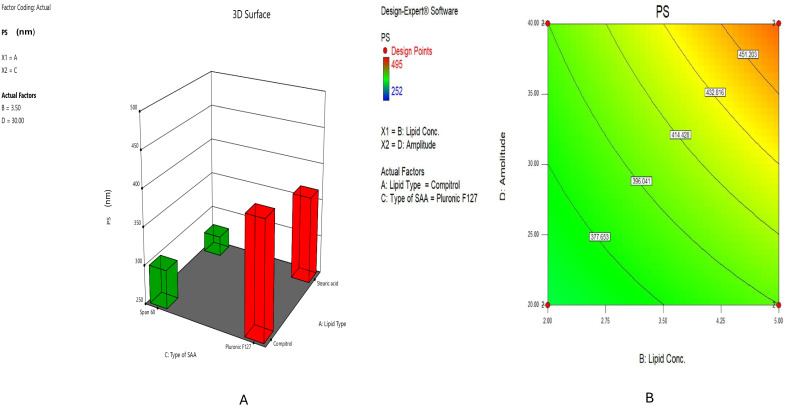
(**A**) 3D surface of the effect of lipid type (A), and type of surfactant (C) on PS; (**B**) contour plot of the effect of lipid conc (B) and sonication amplitude (D) on PS. Abbreviation: PS—particle size, SAA—Surface active agent.

According to ANOVA outcomes, the dependent variables substantially impacted the size of SLNs. The PS was significantly impacted by the solid lipid type (X1) (*p* < 0.0001). As shown in Figure 1A and Table 1, the Stearic acid-based formula, such as HESP-SLN 1, showed a significantly smaller particle size (PS) of 275 ± 2.26 nm compared to the Compritol^®^ ATO 888-based formula, such as HESP-SLN 3, which had a PS of 300.8 ± 5.23 nm; this might be explained by the chemical structure of glyceryl dibehenate, a central component of Compritol^®^ ATO 888, and the prolonged alkyl chain associated with dibehenic acid (C22). In contrast, Stearic acid is characterized by a saturated carbon chain (C18) [35]. Consequently, the choice of lipid excipient is a crucial factor influencing the physical characteristics and stability of SLNs in pharmaceutical formulations [36,37].

Lipid concentration (X2) positively impacted PS (*p* < 0.0001), as depicted in Figure 1B. As the lipid concentration increased, the PS enlarged. It was observed that formulae prepared with 2% lipid concentration, such as HESP-SLN 4 (PS: 267.7 ± 1.89 nm), had a smaller particle size compared to those prepared with 5% lipid concentration, such as HESP-SLN 12 (PS: 284 ± 2.07 nm). These results can be clarified through the fact that SLNs’ PS is closely correlated with the lipid content, which is predicted to affect the tendency to coalesce together at high lipid levels. These findings agreed with Alhakamy et al. [38], who declared the difference between the internal and external phases’ density, which led to lipid flakes forming when the solid lipid concentration was increased. This result corresponded to insufficient surfactant to adequately coat the surface of the particles, which led to the enlargement of particle size [39,40].

Regarding the impact of surfactant type (X3), comparing Pluronic^®^ F127 to Span^®^ 60, the results in Figure 1A indicated that the Pluronic^®^ F127 raised PS (*p* < 0.0001). PS decreased when the HLB value decreased, which is compatible with Rubab et al. [41]; the association between surfactant hydrophobicity and particle size might clarify this result. Specifically, a decrease in surface energy combined with an enhancement in surfactant adsorption area (SAA) causes a rise in hydrophobicity. Therefore, the Span^®^ 60-based formula, such as HESP-SLN 2 (PS: 252 ± 3.12 nm), displayed a significantly smaller particle size compared to the Pluronic^®^ F127-based formula, such as HESP-SLN 6 (PS: 354.4 ± 2.33 nm). This can be ascribed to the lower (HLB = 4.7) of Span^®^ 60, in contrast with the higher (HLB = 22) of Pluronic^®^ F127. The elevated PS referred to the presence of these surfactants in the hydrophilic layer, encouraging water absorption [36].

Concerning sonication amplitude (X4), a significant effect (*p* < 0.0001) value on PS, elevated prop sonication amplitude had directly correlated with a PS augmentation. It was found that increasing the probe sonication amplitude to 40% in HESP-SLN 13 resulted in a larger particle size (383.5 ± 1.26 nm) compared to using 20% probe sonication amplitude, as in HESP-SLN 14, which had a particle size of 370 ± 2.58 nm, as shown in Figure 1B, as higher sonication amplitudes generate intensified shear forces. These shear forces create surface charges, inducing interactive forces that concurrently promote the coalescence of smaller particles into larger aggregates. Hence, elevated PS [42].

#### 2.2.2. Impact of Formulation Variables on PDI

The uniformity of the dispersion is affected by the distribution of particle sizes, which the PDI assesses. It was previously stated that a small particle size distribution is specified by PDI values less than 0.7 [43]. The PDI varied between 0.239 ± 0.012 and 0.571 ± 0.161, as presented in Table 1, implying the limited size distribution, uniformity, and homogeneity of the prepared dispersion [44].

**Figure 2:**
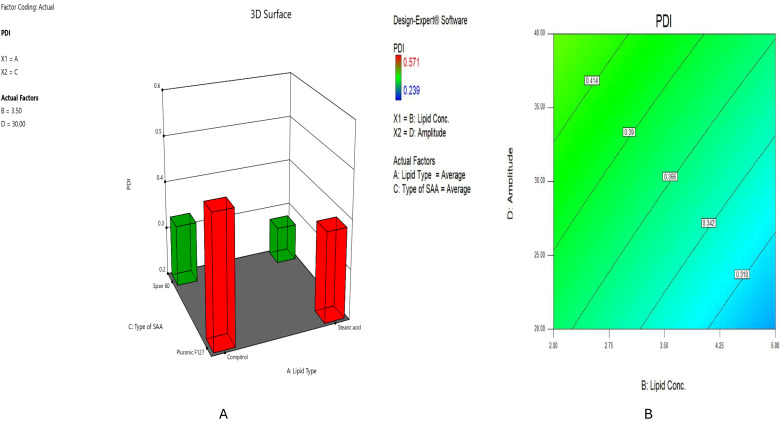
(**A**) 3D diagram of the effect of lipid type (A) and type of surfactant (C) on PDI; (**B**) contour plot of the effect of lipid conc (B) and sonication amplitude (D) on PDI. Abbreviation: PDI—polydispersity index, SAA—Surface active agent.

Accordingly, lipid type (X1) also influenced (*p* < 0.0001) PDI significantly (Figure 2A). Stearic acid-based SLNs, such as HESP-SLN 5, revealed a lower polydispersity index (PDI) value (0.476 ± 0.151) compared to the Compritol^®^-based formula, such as HESP-SLN 7, which had a higher PDI (0.571 ± 0.161). This is mainly due to the consistent crystallization process shown by Stearic acid, resulting in particles with enhanced stability and size uniformity. Nevertheless, the complex nature of Compritol^®^ ATO 888 may lead to irregular crystallization processes, which could contribute to increased variability in nanoparticle size and, hence, increased PDI [45].

Furthermore, lipid concentration (X2) had significantly negatively impacted significantly (*p* < 0.0001) PDI (Figure 2B). Elevating the concentration of lipids decreases the polydispersity index (PDI) of the resultant nanoparticles. HESP-SLN 8, formulated with 2% Compritol^®^ ATO 888, demonstrated a higher polydispersity index (0.492 ± 0.146) compared to HESP-SLN 16, which contained 5% of the same lipid and had a lower PDI (0.378 ± 0.143). High lipid concentrations facilitate an enhanced surface area for the interaction of drug molecules, thereby improving a more homogenous drug distribution within the lipid matrix. This homogeneity reduces the probability of agglomeration and size discrepancies, prevalent challenges in nanoparticle formulation [46,47].

However, surfactant type (X3) was found to have a significant impact (*p* < 0.0001) on PDI, Figure 2A. It can be observed that the formulae prepared with Span^®^ 60, such as HESP-SLN 9, showed a lower polydispersity index (PDI: 0.294 ± 0.132) compared to those prepared with Pluronic^®^ F127, HESP-SLN 13, which indicated a higher PDI (0.334 ± 0.174). This variation might be ascribed to the enhanced lipophilicity nature of Span^®^ 60, with HLB 4.7 relative to Pluronic^®^ F127 with HLB 22; in this case, it is believed that Span^®^ 60 provides high negative charges, which produce the nanoparticles with greater stability compared to Pluronic^®^ F127. Forming smaller and more stable internal phases produces more homogeneous formulae, enhancing stability and contributing to uniform particle size distribution [48].

In addition to (X4), the PDI increased drastically (*p* < 0.0001) as the probe sonication amplitude amplified (Figure 2B). The PDI value for formulae prepared using 40% probe sonication amplitude, such as HESP-SLN 9 (PDI: 0.294 ± 0.132), was greater than those prepared with 20% probe sonication amplitude, such as HESP-SLN 10 (PDI: 0.239 ± 0.012). This elevated energy input can promote the interactional forces of particles that simultaneously endorse aggregation of nanoparticles, resulting in the development of larger aggregates that contribute to the observed higher PDI [49].

#### 2.2.3. Impact of Formulation Variables on ZP of HESP-SLNs

The values of ZP of HESP-SLNs ranged from −22.9 ± 2.12 to −39.4 ± 0.92 mV. As displayed in Table 1, nanoparticles with ZP larger than (−30 mV) have the optimal repulsive force between particles to prevent accumulations, which increases the stability of the particles, according to previous investigations [50]. All HESP-SLNs exhibited a negative ZP potential. This negative charge can be accredited to the partial fatty acid ionization in the glycerides utilized, specifically Compritol^®^ ATO 888, in addition to Stearic acid.

**Figure 3:**
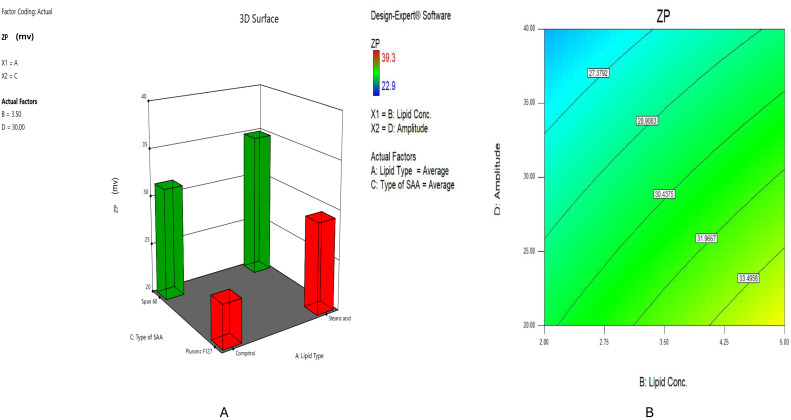
(**A**) 3D diagram of the effect of lipid type (A), and type of surfactant (C) on ZP; (**B**) contour plot of the effect of lipid conc (B) and sonication amplitude (D) on ZP. Abbreviation: ZP—zeta potential, SAA—Surface active agent.

Therefore, X1: solid lipid type (*p* < 0.0001) significantly influenced ZP, Figure 3A, where Stearic acid-based formulae, such as HESP-SLN 13 (ZP: −29.4 ± 3.79 mV), displayed the highest negative zeta potential (ZP) compared to the Compritol^®^ ATO 888-based formulae, such as HESP-SLN 15 (ZP: −24.4 ± 1.74 mV). Such a result might be referred to as the negative charge of the carboxylic acid moiety (C_17_H_35_COOH) on Stearic acid concentrated on the nanoparticles’ surface [51]. This negative charge promotes electrostatic repulsion among particles, thus keeping the nanoparticle dispersion more stable over a long time. Furthermore, the negatively charged surface of SLNs prevents particle aggregation and enables their interaction with positively charged molecules, thus possibly enabling targeted drug delivery applications [52].

The effect of increasing the lipid concentration (X2) on the ZP of HESP-SLNs is shown in Figure 3B. It was found that the zeta potential (ZP) of HESP-SLN 2, prepared with a 2% concentration of Stearic acid, was −36.2 ± 1.52 mV, while HESP-SLN 10, prepared with a 5% concentration of the same lipid, exhibited a ZP of −39.4 ± 0.92 mV. As the concentration of these lipids increases, the quantity of negative charge groups on the nanoparticle surface also rises, enhancing the overall negative zeta potential. This higher charge contributes to increased electrostatic repulsion between particles, promoting colloidal stability and demonstrating that lipids significantly impact the negative charge level on the surface of nanoparticles [53,54].

Moreover, the surfactant type (X3) significantly affected the ZP values (*p* < 0.0001), Figure 3A. The HESP-SLNs prepared with Span^®^ 60 proved a significantly more negative zeta potential (ZP), with HESP-SLN 1 showing a ZP of −31.7 ± 2.47 mV, compared to the Pluronic^®^ F127-based formulae, such as HESP-SLN 5, which demonstrated a lower ZP of −25.3 ± 2.59 mV, which could be implied by an increase in hydroxide ion concentration providing highly negative charges, thereby enhancing the stability of the nanoparticle compared to that achieved with Pluronic^®^ F127. This may be ascribed to the comparatively greater hydrophilicity of Pluronic^®^ F127, which possesses fewer lipophilic repeating units and has an HLB of 22, in contrast to Span^®^ 60, which has an HLB of 4.7, providing a negatively charged shield on the surface of the vesicular bilayer, thereby enhancing the stability of HESP-SLNs [48].

The sonication amplitude (X4) had a significant impact on the ZP values (*p* < 0.0001), as shown in Figure 3B. It was noted that HESP-SLNs-3 with 40% of a sonication amplitude and HESP-SLNs-4 with 20% provide ZPs of −23.5 ± 4.25, −35.4 ± 3.53 mV, respectively, where employing 20% of a sonication amplitude provides an adequate level of energy to generate a fine homogenous dispersion of particles, preventing the risk of inducing excessive heat or shear stress that may lead to the destabilization of the nanoparticles [55]. Certainly, this confirmed the high negative ZP value of HESP-SLNs, indicating competent colloidal stability and preventing aggregation resulting from strong electrostatic repulsion between particles.

#### 2.2.4. Impact of Formulation Variables on EE% of HESP-SLNs

**Figure 4:**
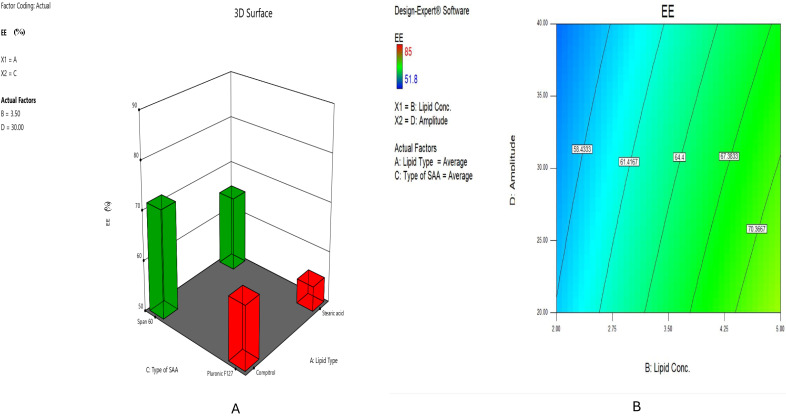
(**A**) 3D diagram of the effect of lipid type (A) and type of surfactant (C) on EE; (**B**) contour plot for the effect of lipid conc (B) and sonication amplitude (D) on EE. Abbreviation: EE—entrapment efficiency, SAA—Surface active agent.

As displayed in Table 1, The percentage of encapsulation efficiency fluctuated from 51.8 ± 0.86% to 88.2 ± 2.09%. Figure 4A demonstrated the EE percentage of the formulae was considerably (*p* < 0.0001) impacted by the type of lipid (X1), Compritol^®^ 888 ATO-based formulae, such as HESP-SLN 12, indicating an encapsulation efficiency (EE) of 85.1 ± 2.12%, which was lower than that of Stearic acid-based formulae, such as HESP-SLN 10, which showed a higher EE of 88.2 ± 2.09%. This was due to its complicated composition, which resulted from a long-chain fatty acid (C22) associated with mono-, di-, and triacylglycerols causing an imperfect orientation. This allowed for greater space for the loaded drug and improved the drug’s solubility and incorporation into the lipid [35]. These outcomes showed a high correlation with PS findings.

The lipid concentration (X2) revealed a significant effect (*p* < 0.0001) on encapsulation efficiency, Figure 4B, suggesting that a rise in lipid content might raise the encapsulation efficiency of hydrophobic drugs such as hesperidin. The encapsulation efficiency (EE) percentages of HESP-SLN 7, formulated with 2% Compritol^®^ ATO 888, and HESP-SLN 15, formulated with 5% of the same lipid, were 58.6 ± 1.13% and 64.2 ± 1.34%, respectively. This could be explained by the low lipid concentration, which is insufficient for entrapping the hydrophobic drug. However, a greater concentration might entrap the available drug and result in the highest EE%, thus decreasing the probability of drug leakage during the nanoparticle formation process [56].

Additionally, in Figure 4A, the EE% data showed that the X3: Type of SAA had considerable (*p* < 0.0001) influence on the encapsulation efficiency (EE%) of HESP-SLN 9, prepared using Span^®^ 60, which was 72.4 ± 1.7%, while HESP-SLN 13, prepared using Pluronic^®^ F127, showed a lower EE of 55.8 ± 1.36%, which indicated that a decrease in the HLB level of the used SAA improved the EE%. Despite a low HLB of 4.7, Span^®^ 60 was effective in entrapping much more of the hydrophobic drugs in the SLNs’ core, resulting in the greatest encapsulation efficiency percentage (EE%) [57], in contrast with Pluronic^®^ F127, which had high HLB values of 22 with high hydrophilic properties.

Concerning the impact of sonication amplitudes (X4), higher amplitudes of 40% have a negative impact on the EE%, as depicted in Figure 4B, where HESP-SLNs-3 with 40% amplitude demonstrated an EE% of 59.2 ± 2.14, while the HESP-SLNs-4 with 20% amplitude displayed an EE% of 65.8 ± 3.02. This is possibly because of the potential to induce excessive cavitation and shear forces, which can disturb the SLNs formulae and the possibility of drug leakage from the nanoparticle, hence decreasing encapsulation efficiency [42].

### 2.3. Identifying the Optimized HESP-SLNs

**Figure 5:**
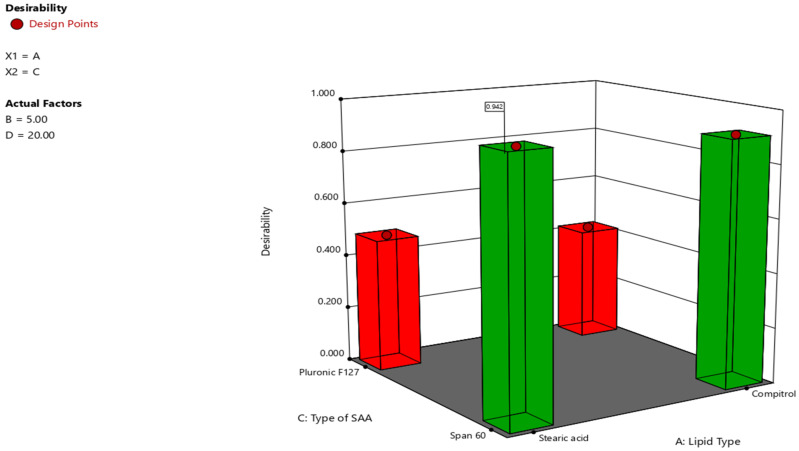
3D diagram for the desirability of the optimized HESP-SLNs 10. Abbreviations: HESP—hesperidin; SLNs—solid lipid nanoparticles, SAA—Surface active agent.

The desired constraints were chosen to maximize EE% and ZP, as well as minimize PS and PDI; therefore, HESP-SLNs10 possessed an EE of 88.2 ± 2.09%, a ZP of −39.4 ± 0.92 mV, a PS of 280 ± 1.35 nm, and a PDI of 0.239 ± 0.012 was selected as the optimized formula (desirability = 0.942). Figure 5 contains Stearic acid as a lipid, Span^®^ 60 as an SAA, 5% lipid concentration, and 20% amplitude sonication [58]. Specifically, a strong correlation between the predicted and observed values was found. Consequently, the HESP-SLNs10 optimized formulae require additional investigation.

### 2.4. Improvement of Optimized HESP-SLNs Properties Producing Essential Oil-Loaded HESP-NLCs

#### 2.4.1. Screening and Selecting Essential Oils

##### Agar Diffusion Assay

The essential oils utilized during the current study were evaluated for antibacterial activity by determining the radius of the zone of inhibition, followed by the strains being incubated under ideal conditions; the provided results of the radius values of the zone of inhibition in Table 3 display that the essential oil (TTO) has the largest diameter of inhibition zone amongst all the essential oils, with 35 ± 2.5 mm of *E. coli*, 37 ± 2 mm of *P. aeruginosa*, and 45 ± 2 mm of *S. aureus*. This result complied with Dalal et al. [59] who stated that TTO demonstrated superior antibacterial activity and a high ability to function as an antiseptic property, which could be due to its hydrocarbon structure and lipophilicity, which enabled it to partition into biological membranes, hindering their essential functions selectively. Therefore, TTO is considered an excellent treatment for open wounds and sores.

#### 2.4.2. Evaluation of the Minimum Inhibitory Concentration (MIC)

**Figure 6:**
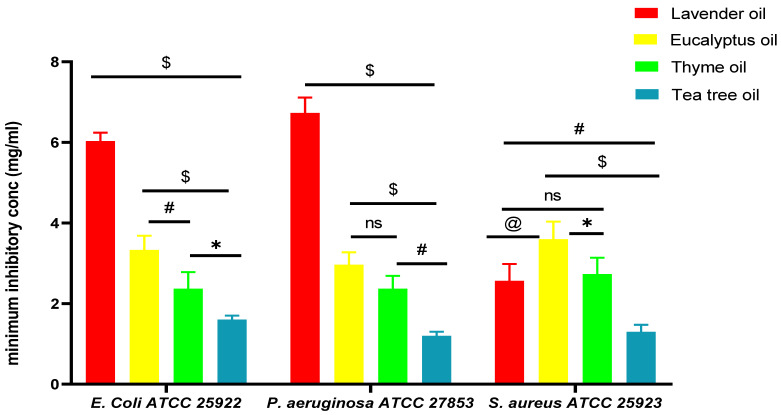
Minimum inhibitory concentration (MIC) of essential oils(mg/mL). Data are reported as the average ± standard deviation obtained from three independent trials (n = 3). Statistical analysis was carried out using one-way ANOVA followed by Tukey’s Multiple Comparisons test. (ns) = not significant; ($) = *p* < 0.0001; (#) *p* < 0.001; (@) = *p* < 0.01; (*) = *p* < 0.1. Abbreviations: *E. coli*—*Escherichia coli*; *P. aeruginosa*—*Pseudomonas aeruginosa*; *S. aureus*—*Staphylococcus aureus*.

As indicated in Table 4 and Figure 6, the MIC was measured to determine the antibacterial efficacy of essential oils. TTO was shown to have potent antibacterial activity against both Gram-positive and Gram-negative bacteria, among the other essential oils examined. The demonstrated MIC of TTO was 1.6 ± 0.1 mg/mL of *E. coli*, 1.2 ± 0.2 mg/mL of *P. aeruginosa*, and 1.2 ± 0.3 mg/mL of *S. aureus*. This may be caused by terpinen-4-ol, which possesses the same ability as various disinfectants and preservatives, such as phenol derivatives, to deteriorate proteins and change the structure and function of cell wall membranes, which leads to disruption of the cytoplasmic membrane of the bacterial cell causing bacterial cell death [60]. These results agreed with a former study that described subjecting these organisms to TTO at the corresponding MIC, which consequently permeated the cell walls and cytoplasmic membranes and caused the loss of their structural integrity and function. This allowed intracellular substances to escape, preventing them from retaining homeostasis and preventing respiration [61].

### 2.5. Investigation of HESP-NLCs

The average diameter of particles (PS), polydispersity index (PDI), and zeta potential (ZP) of the formed HESP-NLCs were assessed utilizing a Zeta sizer device, which provided the following values: PS of 300 ± 5.21 nm, PDI of 0.272 ± 0.023, as well as ZP of −39 ± 0.42, where the encapsulation efficacy was 93.7 ± 1.55%.

### 2.6. Transmission Electron Microscopy (TEM) of the Optimized HESP-SLNs and HESP-NLCs

**Figure 7:**
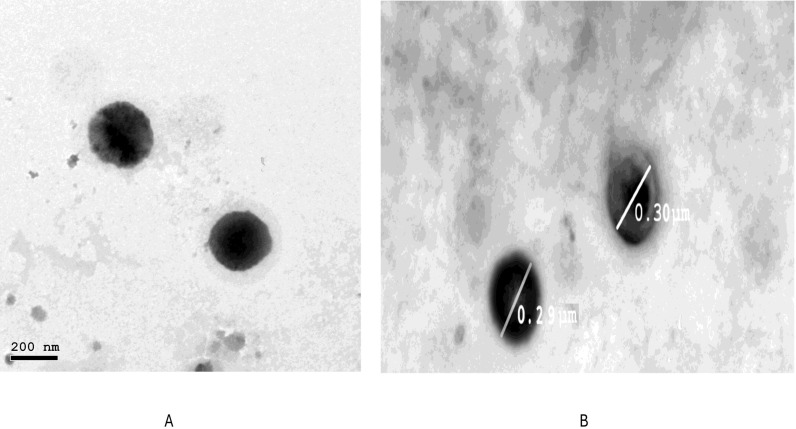
Transmission electron microscopy of the optimized HESP-SLNs (**A**) and HESP-NLCs (**B**). Abbreviations: HESP—hesperidin; SLNs—solid lipid nanoparticles, NLCs—manostructured lipid carriers.

The morphological examination of HESP-SLNs and HESP-NLCs was established by TEM examination, as illustrated in Figure 7. The nanoparticles were uniformly sized and had spherical shape without accumulation [62]. Consequently, the average PS obtained by the Zeta sizer perfectly correlated with the TEM findings.

### 2.7. Compatibility Evaluation Optimized HESP-SLNs and HESP-NLCs

#### 2.7.1. Differential Scanning Calorimetry (DSC)

**Figure 8:**
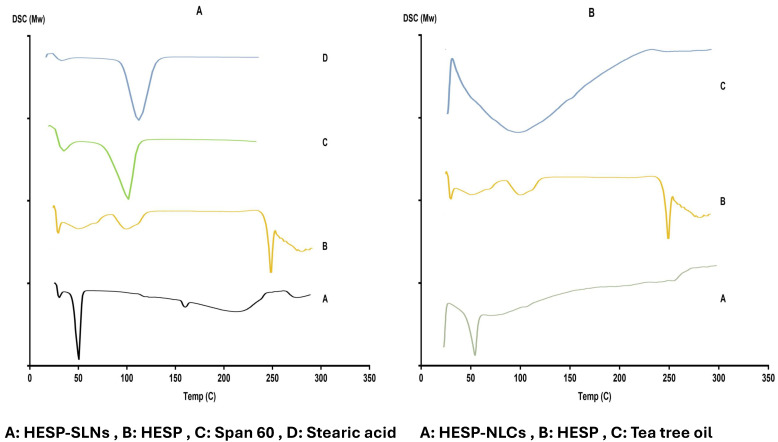
Differential scanning calorimetry of HESP-SLNs (**A**) and HESP-NLCs (**B**). Abbreviations: HESP—hesperidin; SLNs—solid lipid nanoparticles; NLCs—nanostructured lipid carriers.

DSC analysis (Figure 8A,B) is a vital tool for understanding the thermal behavior of HESP, as well as its formulations in SLNs and NLCs [63]. By assessing changes in thermal events, DSC provides insights into drug encapsulation efficiency, crystallinity alterations, and drug–lipid interactions, all of which critically influence formulation stability, drug release, and storage properties [64]. The thermogram of pure HESP displays a sharp endothermic peak at 257.74 °C, corresponding to its melting point, consistent with the literature reports [65,66]. This peak confirms the highly crystalline nature of HESP. However, this characteristic peak is completely absent in the thermograms of HESP-SLNs and HESP-NLCs, indicating that HESP is molecularly dispersed within the lipid matrix or transformed into an amorphous state. Such a transformation enhances the dissolution properties and bioavailability of HESP, which are essential for effective therapeutic delivery. The lipid components, Stearic acid (melting at 57.32 °C) and Compritol^®^ 888 ATO (melting at 60.24 °C), exhibit sharp, distinct melting peaks in their pure forms, confirming their crystalline structure. In HESP-SLNs and HESP-NLCs, these peaks shift to lower temperatures and broaden significantly, particularly in NLCs. This broadening and shift signify a partial loss of crystallinity due to molecular interactions between HESP and the lipid components, as well as structural reorganization influenced by tea tree oil in the NLCs. These interactions result in decreased lattice energy and reduced thermal stability, highlighting the amorphous nature of the lipid–drug matrix. A significant difference in melting enthalpy (ΔH) is noted between SLNs and NLCs. SLNs, being fully composed of solid lipids, demonstrate higher ΔH values, indicative of a more ordered crystalline structure.

In contrast, NLCs exhibit considerably lower ΔH, reflecting a greater degree of disorder within their lipid matrix. This reduction in crystallinity in NLCs is attributed to the incorporation of tea tree oil, which disrupts lipid packing and creates imperfections in the solid lipid lattice. This disruption enhances the drug loading capacity of NLCs, minimizes the risk of drug expulsion during storage, and promotes sustained drug release. Amorphous or molecularly dispersed drugs generally exhibit slower and more controlled release kinetics, reducing the likelihood of burst release effects. Additionally, the absence of any new exothermic or endothermic peaks in the thermograms confirms that no significant chemical degradation, polymorphic transitions, or drug–lipid complex formations occur during encapsulation. This finding underscores the physical stability of HESP within the lipid formulations and the absence of unfavorable drug–lipid interactions. Overall, DSC analysis demonstrates the successful encapsulation of HESP within SLNs and NLCs, with the drug existing in a non-crystalline, amorphous state. The reduced crystallinity in NLCs further supports their superior drug loading capacity, enhanced physical stability, and prolonged release, making them a promising formulation for improved bioavailability and therapeutic efficacy.

#### 2.7.2. Raman Spectroscopy

**Figure 9:**
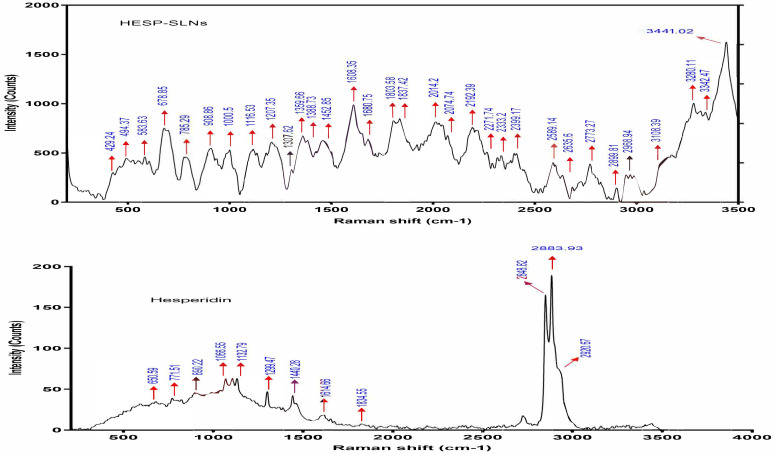
Raman spectroscopy of pure HESP powder and HESP-SLNs. Abbreviations: HESP—hesperidin; SLNs—solid lipid nanoparticles.

Raman spectroscopy is a powerful, non-destructive analytical technique capable of providing high-resolution insights into molecular interactions and structural modifications. Its sensitivity to functional group vibrations enables precise detection of changes in the molecular environment of HESP upon encapsulation into SLNs and NLCs. The Raman spectrum of pure HESP (Figure 9) exhibits distinct and well-defined peaks, which correspond to specific vibrational modes of its functional group assignments according to the literature [67,68]. The Raman spectrum of pure HESP (Figure 9) exhibits distinct and well-defined peaks, which correspond to specific vibrational modes of its functional groups. Peaks at 2921 cm^−1^, 2884 cm^−1^, and 2849 cm^−1^ are attributed to aromatic C–H stretching vibrations, while the strong peak at 1647 cm^−1^ reflects C=O stretching vibrations characteristic of the flavonoid backbone. The peak at 1614 cm^−1^ is assigned to aromatic ring stretching in rings A and B. Additional features, such as the peak at 1440 cm^−1^, are attributed to C–OH stretching coupled with vibrations in ring A, while the peak at 1300 cm^−1^ corresponds to ring B stretching, combined with C–C and O–H bending vibrations. Peaks in the 1060–1130 cm^−1^ range are attributed to glycosidic ring breathing modes, while the band at 771 cm^−1^ is associated with out-of-plane bending of C–H, as well as C–O–C and C–C–C bending within ring A. These assignments align with previously reported Raman spectra of flavonoids, ensuring a reliable interpretation of each peak. Upon encapsulation into SLNs, significant spectral changes are observed (Figure 9), including the attenuation or disappearance of key peaks, such as those at 2921 cm^−1^, 2884 cm^−1^, 1647 cm^−1^, 1614 cm^−1^, 1300 cm^−1^, 1159 cm^−1^, and 771 cm^−1^. Notably, the intensity of the 1647 cm^−1^ C=O stretching peak decreases by approximately 60%, indicating strong molecular interactions between HESP and the solid lipid matrix. This reduction in peak intensity suggests the restricted vibrational freedom of HESP molecules due to encapsulation, likely caused by physical confinement and hydrogen bonding between the hydroxyl groups of HESP and polar functional groups (e.g., hydroxyl or ester groups) in the lipid matrix. The disappearance of glycosidic ring breathing vibrations (1060–1130 cm^−1^) and aromatic ring bending (771 cm^−1^) further supports the hypothesis of the deep embedding of HESP within the solid lipid matrix, leading to molecular shielding and alterations in energy levels.

**Figure 10:**
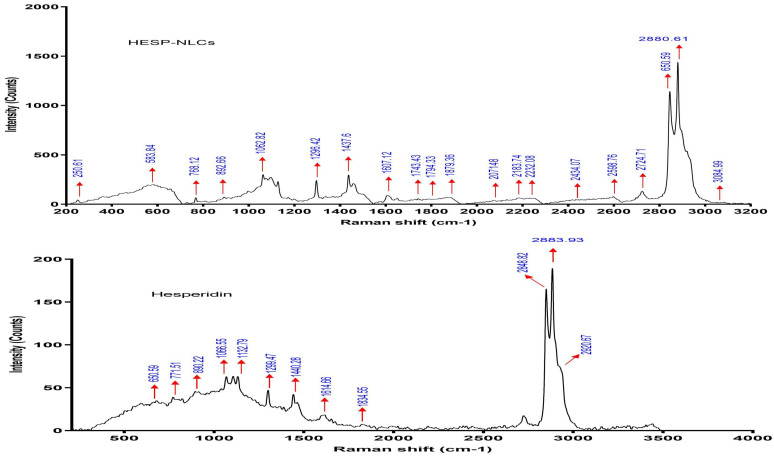
Raman spectroscopy of pure HESP powder and HESP-NLCs. Abbreviations: HESP—hesperidin; NLCs—nanostructured lipid carriers.

In contrast, the Raman spectrum of HESP-loaded NLCs (Figure 10) reveals distinct features that differentiate it from both pure HESP and SLNs. The retention of some HESP peaks, such as the 2880 cm^−1^ peak, and the emergence of a new peak at 3084 cm^−1^, unique to the NLC spectrum, suggest the presence of the liquid lipid component (tea tree oil). This component provides a more dynamic and flexible encapsulation environment. Additionally, shifts in glycosidic ring breathing modes (1060–1130 cm^−1^) and aromatic C–H stretching vibrations (from 2921 cm^−1^ to 2880 cm^−1^) indicate altered molecular packing in the NLC matrix. The partial reduction in the 1647 cm^−1^ C=O stretching peak intensity (approximately 30% reduction compared to pure HESP) further supports the conclusion that HESP experiences less restrictive interactions in NLCs compared to SLNs. This difference is attributed to the amorphous nature of the liquid lipid component in NLCs, which allows for greater molecular mobility and more dynamic encapsulation. Overall, these spectral findings confirm the optimal encapsulation of HESP in both lipid matrices. The SLNs, with their rigid crystalline structure, effectively confine HESP molecules, offering strong interactions and restricted mobility that suggest a potential for prolonged and controlled drug release. On the other hand, NLCs, characterized by their amorphous and flexible matrix, provide a less restrictive environment, facilitating higher molecular mobility and faster drug release. Together, these results highlight the distinct advantages of SLNs and NLCs as lipid-based drug delivery systems with encapsulation profiles tailored to specific therapeutic needs [69].

### 2.8. In Vitro Release of Optimized HESP-SLNs and HESP-NLCs

**Figure 11:**
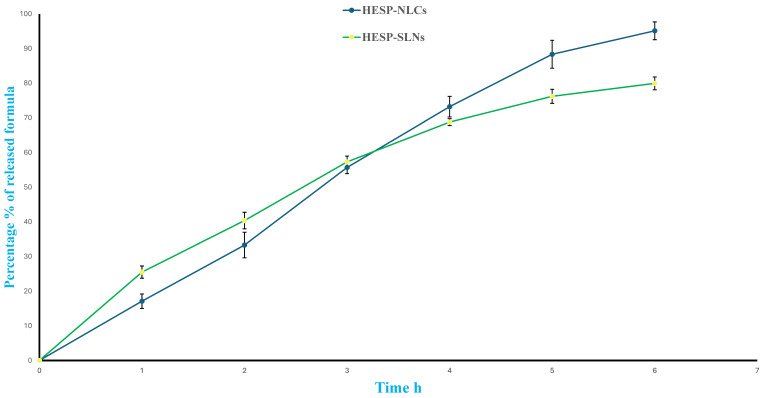
In vitro drug release of HESP-SLNs and HESP-NLCs. Data are reported as the average ± standard deviation obtained from three independent trials (n = 3). Abbreviations: HESP—hesperidin; SLNs—solid lipid nanoparticles; NLCs—nanostructured lipid carriers.

The release profile of HESP was 95.14 ± 3.45% and 79.96 ± 2.89% for HESP-NLCs and HESP-SLNs after 6 h, respectively. As depicted in Figure 11, HESP-SLNs displayed a primary burst release within the initial hour, a constant pattern subsequently, and sustained release over the next few hours. This could probably be related to the amount of HESP that could adsorb on the outer layer of the nanoparticle or possibly cause the particle-matrix to separate, which dissolves immediately, providing spontaneous therapeutic effects while simultaneously maintaining consistent drug release to sustain therapeutic concentrations over a prolonged period [70].

Notably, HESP-NLCs achieved a higher Q6%, 95.14%, compared to the Q6% of 79.69% for HESP-SLNs. This impact was explained by the different physical states of the lipids in the two types of nanoparticles. Where both liquid and solid lipids are incorporated into the HESP-NLCs, the NLCs’ structure becomes more imperfect, which improves drug loading efficiency; whereas, in HESP-SLNs, the lipids are more densely and ordinately packed, therefore delaying the drug release, while the less orderly and less crystalline structure of NLCs can comparatively accelerate drug release [71]. Multiple release kinetic models were fitted with release data, particularly the zero-order, first-order, and Higuchi, in addition to Korsmeyer–Peppas models; regarding the HESP-SLNs, a direct relationship was identified that fitted perfectly Higuchi’s model, owing to the highest regression coefficient R^2^ (0.983), which revealed that drugs are released by diffusion from the SLNs dispersion. These findings concurred with prior investigations that reported the diffusion mechanism of SLNs formulae [72], whereas for HESP-NLCs, the highest R² value (i.e., 0.9872) suggested the best-fit model was Korsmeyer–Peppas, and the release exponents (n) were found to be 0.9047, implying the non-Fickian or nature of diffusion from the formulation (1 > n > 0.50) [73].

### 2.9. Storage Stability Studies

The optimized HESP-SLNs and HESP-NLCs were maintained at 25 °C and 4 °C for 3 months. They had no vesicle aggregation or precipitation during storage, nor displayed any apparent color or odor alterations. A key factor in predicting and preventing particle aggregation is the zeta potential, which is a measure of the surface charge of nanoparticles. High zeta potential nanoparticles, often greater than ±30 mV, are shown to have considerable electrostatic repulsion, which substantially reduces their tendency to aggregate. Van der Waals forces, which seek to attract particles together more closely, are counteracted by this repulsion [74]. The measured zeta potential helps in the development of a charged layer surrounding the nanoparticles and offers details regarding the efficacy of stabilizing substances employed in SLNs, and NLCs formulations. As presented in Table 5 and Table 6, a zeta potential value greater than −30 mV throughout a 3-month period is a sign of optimized HESP-SLNs, and HESP-NLCs stability; potential attraction forces that can cause particle aggregation are successfully counteracted by electrostatic forces when a high negative zeta potential is maintained over an extended length of time [75,76]. In particular, the PS, ZP, PDI, and EE % were statistically evaluated, revealing that the outcomes of the stored formulae did not vary significantly (paired *t*-test, *p* > 0.05) from the formula that was freshly prepared, representing that both the optimized HESP-SLNs and HESP-NLCs showed stability when maintained at 4 °C and 25 °C over three months. This result corresponded to various research investigations demonstrating lipid-based nanoparticles’ enhanced stability, specifically SLNs and NLCs [77].

### 2.10. Determination of In Vitro Antibacterial Properties

**Figure 12:**
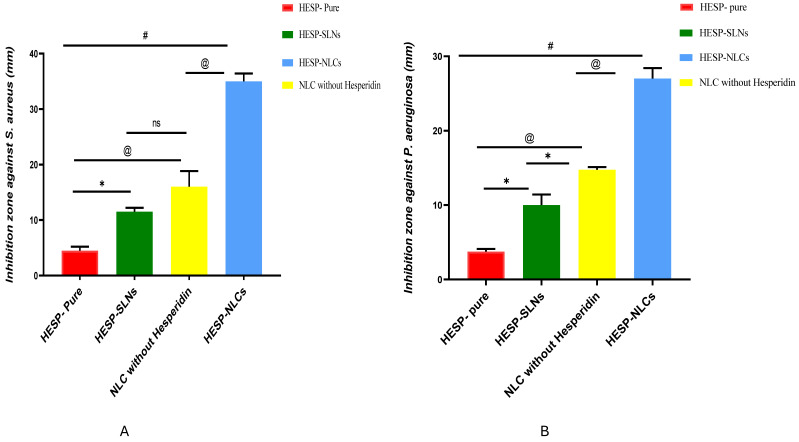
(**A**) The inhibition zone (mm) of pure HESP, HESP-NLCs, HESP-SLNs, and NLCs-without HESP against Gram-positive Staphylococcus aureus; (**B**) the inhibition zone (mm) of pure HESP, HESP-NLCs, HESP-SLNs, NLCs-without HESP against Gram-negative Pseudomonas aeruginosa. Data are reported as the average ± standard deviation obtained from three independent trials (n = 3). Statistical analysis was carried out using one-way ANOVA followed by Tukey’s Multiple Comparisons test. (ns) = not significant; (#) = *p* < 0.001; (@) = *p* < 0.01; (*) *p* < 0.1. Abbreviations: HESP—hesperidin; SLNs—solid lipid nanoparticles, NLCs—nanostructured lipid carriers; *S. aureus*—*Staphylococcus aureus*; *P. aeruginosa*—*Pseudomonas aeruginosa*.

Antibacterial activity was evaluated by comparing various formulae, including HESP-SLNs, HESP-NLCs, NLCs without HESP, and a suspension of pure HESP (1% in distilled water) as a control. As shown in Figure 12A, the inhibition zone of these formulae against Gram-positive *Staphylococcus aureus* (ATCC 25923), and in Figure 12B the inhibition zone of these formulae against Gram-negative *Pseudomonas aeruginosa* (KCTC 2513) were statistically different (*p* < 0.0001). It was observed that HESP-SLNs demonstrated a substantial antibacterial impact (*p* < 0.05) when compared with pure HESP, where the inhibition zones against the *S. aureus* and *P. aeruginosa* were greater than pure HESP, by 2.7-fold and 2.5-fold individually.

On the contrary, NLCs without HESP displayed a significant decline (*p* < 0.01) in bacterial proliferation when compared with pure HESP; the inhibition zones against *S. aureus* and *P. aeruginosa* were larger than pure HESP, by 3.5-fold, and 3.8-fold, respectively. However, there is a non-significant difference when compared to HESP-SLNs, which was definitively associated with the antibacterial characteristics of TTO. This outcome agreed with Elsewedy et al. [78], who have previously reported that TTO possesses significant antimicrobial efficacy against various pathogens of bacterial species caused by the main active ingredient of TTO, which is essentially terpinen-4-ol. terpinen-4-ol was attributed to the activation of NF-kB factor, which increases phagocytic activity, differentiation of immature myelocytes into active phagocytizing monocytes, and increases the expression of CD11b. This receptor is partially responsible for the phagocytosis of opsonized bacteria and fungi by leukocytes [79].

On the other hand, HESP-NLCs produced the significantly largest inhibition zones in comparison with other formulae (*p* < 0.05); the inhibition zones against *S. aureus* and *P. aeruginosa* were correspondingly greater by 7-fold and 6-fold when compared with pure HESP, by 2.9-fold and 2.7-fold when compared with HESP-SLNs, and 2.1-fold and 1.8-fold in contrast with NLCs without HESP. Consequently, HESP-NLCs displayed the utmost antibacterial impact on the cultivated bacteria, owing to the synergism effect of the incorporated tea tree oil in HESP-NLCs, leading to superior antimicrobial effectiveness compared to HESP-SLNs and pure HESP [80]. Hence, the result suggested that prepared TTO-loaded HESP-NLCs were a promising formula with superior therapeutic efficacy and antimicrobial activity against several pathogens [81].

### 2.11. Cytotoxicity Assay

A precise in vitro cytotoxicity investigation was conducted to verify formulation safety before the in vivo assessment. The SRB colorimetric assay was considered an advanced method for in vitro cytotoxicity evaluation tests, particularly for evaluating the natural compound’s activity. This method offers superior reproducibility and linearity, reduced susceptibility to environmental fluctuations, and is independent of intermediate metabolism. Furthermore, the SRB colorimetric assay enables the measurement of cell viability by assessing cellular protein content, thus establishing itself as a dependable tool for evaluating the cytotoxic impact of various formulae [82].

The fibroblast plays a crucial role in the generation of collagen and the creation of extracellular matrix; moreover, it contributes to the secretion and assembly of matrix components required for cell migration, provides signals for re-epithelialization, produces bioactive mediators promoting cellular differentiation and tissue regeneration, and regulates the wound-healing process [83]. Because Human skin fibroblast (HSF) cells are an excellent in vitro model for tracking the processes occurring in human skin and studying the potential impact of various biologically active substances on these processes, they were selected as an appropriate in vitro model for assessing how TTO-loaded Hesperidin-NLCs affect cellular responses that are essential for wound healing [84]. A similar cell line was selected to conduct a Cytotoxicity Assessment by Pornpitchanarong et al. [85].

**Figure 13:**
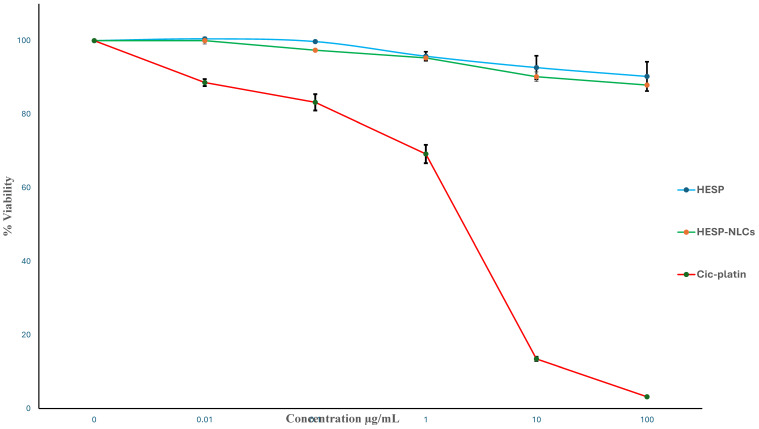
In vitro cytotoxicity test. Data are reported as the average ± standard deviation obtained from three independent trials (n = 3). Abbreviations: HESP—hesperidin; NLCs—nanostructured lipid carriers.

Since the HESP-NLCs were intended for topical application, as well as the assessment of cytotoxicity using cultured cells of human skin, they are an effective method for evaluating skin damage. Therefore, the HESP pure drug and HESP-NLCs formula demonstrated 87% cell viability in comparison to untreated HSF cells and showed non-considerable toxicity in HSF cells despite high doses (100 ug/mL), with an IC_50_ value exceeding 100 ug/mL, as proved in Figure 13. These findings agreed with a former study [86], which specified the cytotoxicity that depends on the concentration of TTO on dermal fibroblast cells. The in vitro cytotoxicity (percentage of cell viability) of the HESP-NLCs, pure HESP, and the positive control (Cis-Platin) was assessed. There were no significant alterations in the cell viability percentage of the HESP-NLCs compared to each concentration of drug solution (unpaired *t*-test, *p* > 0.05). These outcomes verify the safety behavior of TTO-incorporated HESP-NLCs for topical application on the skin.

### 2.12. In Vivo Study

#### 2.12.1. Assessment of the Percentage of Wound Closure

**Figure 14:**
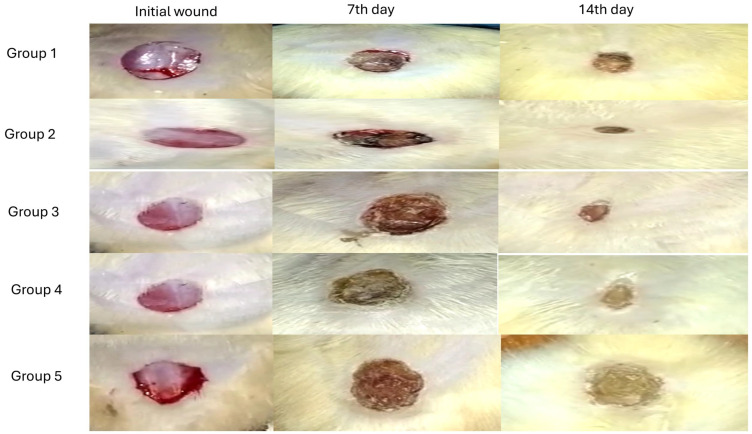
Photographs showing wound closure in different groups after 7 and 14 days. Group 1—SLNs group, Group 2—NLCs group, Group 3—NLCs without drug group, Group 4—HESP pure drug group, Group 5—positive control group. Abbreviations: HESP—hesperidin; SLNs—solid lipid nanoparticles, NLCs—nanostructured lipid carriers.

**Figure 15:**
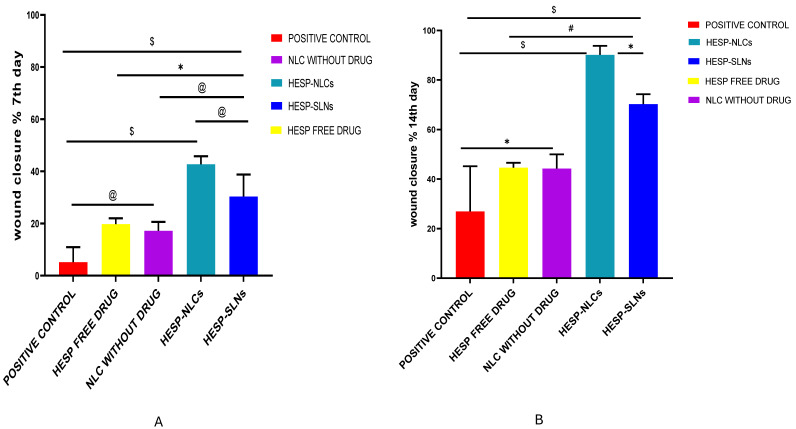
(**A**) Assessment of wound closure percent on day 7, and (**B**) assessment of wound closure percent on day 14 in all groups. Data are reported as the average ± standard deviation obtained from three independent trials (n = 3). Statistical analysis was carried out using one-way ANOVA followed by Tukey’s Multiple Comparisons test. ($) = *p* < 0.0001; (#) *p* < 0.001; (@) = *p* < 0.01; (*) = *p* < 0.1. Abbreviations: HESP—hesperidin; SLNs—solid lipid nanoparticles; NLCs—nanostructured lipid carriers.

Wound healing is a complex and dynamic process that is essential for the recovery and renewal of skin, as well as any damage (chemical or physical). In general, this process can be divided into three stages: (i) inflammation and hemostasis; (ii) proliferation with ECM deposition; and (iii) re-epithelization with tissue remodeling [87]. This study determined the ability of prepared HESP-SLNs and TTO-loaded HESP-NLCs to promote wound healing in wounded rats. As shown in Figure 14 and Figure 15A, on day 7, it was apparent that the wound closure % obtained by GP1 treated with HESP-SLNs and GP2 treated with HESP-NLCs was much faster than the other groups, 30.36% and 42.70%, respectively, where statistical analysis presented a significant variation (*p* < 0.05) among both treated groups. However, the ANOVA results showed there was a substantial rise in the percentage of wound closure in the HESP-NLCs-treated group (GP2) (*p* < 0.0001) in contrast to the (GP5) positive control group and other groups: NLCs without the drug (GP3), and HESP pure drug (GP4).

HESP-SLNs (GP1) displayed a significant enhancement in the percentage of wound closure when contrasted with the positive control (GP5) (*p* < 0.0001), NLCs without HESP (GP3) (*p* < 0.1), as well as in contrast to HESP pure drug (GP4) (*p* < 0.05). Additionally, NLCs without the drug (GP3) showed a substantial variation (*p* < 0.05) from the (GP5) positive control and HESP pure drug (GP4).

On the 14th day of the study, despite the rise in wound closure % detected across all groups in comparison to day 7, GP3, GP4, and GP5 showed signs of inflammation and delayed wound closure, suggesting insufficient healing. However, the HESP-NLCs-treated group (GP2) conveyed a greater percentage of constriction of the wound when compared with HESP-SLNs (GP1), Figure 15B; therefore, HESP-NLCs showed clear superiority, with a significant difference of *p* < 0.05. This could be explained by the complementary and synergistic effect of tea tree oil and hesperidin, which produced an impressive improvement in the percentage of wound closure. TTO is an essential oil that possesses numerous pharmacological actions, such as immunomodulatory, antioxidant, antibacterial, and anti-inflammatory, corresponding to the presence of a combination of terpenes, lipids, and aromatic compounds. Its major constituents include cycloolefins and enol derivatives, such as γ-terpinene, terpinen-4-ol, α-terpinene, α-terpineol, and 1,8-cineole [88]. Terpinen-4-ol prevents the production of inflammatory mediators by monocyte activation, and it helps regenerate collagen [89,90]. In comparison, hesperidin controls several biological functions that promote wound-healing processes, such as cell division, proliferation, death, plasticity, and migration. Hesperidin boosts angiogenesis, which is considered a crucial process that supports the proliferation of new organs and tissue while also providing wounds with nutrients and new cells, promoting the growth of fibroblasts, and keratinocytes, enhancing the synthesis and deposition of collagen, and remodeling the new extracellular matrix, essential for re-epithelialization and faster wound contraction [91,92]; this particularly suggests that the TTO-loaded HESP-NLCs have spectacular potential for additional improvement as a beneficial therapeutic compound promoting wound healing.

#### 2.12.2. Histopathology Study

**Figure 16:**
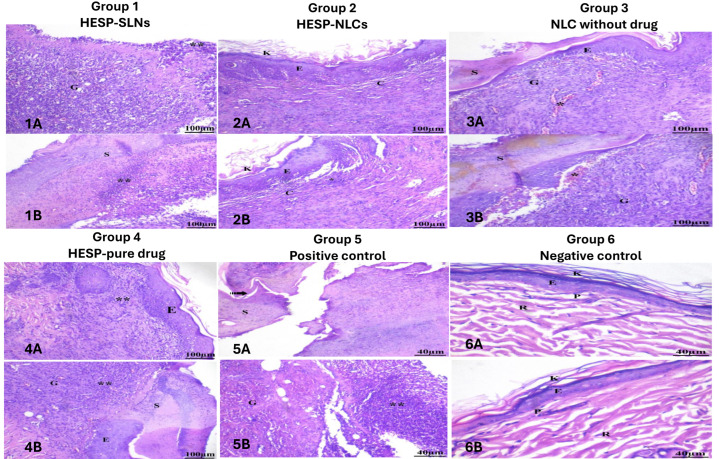
Photomicrographs represent the histopathological differences in skin tissues among examined groups after 14 days (Hematoxylin and Eosin stain), Group 1 (1A, 1B) for HESP-SLNs group, Group 2 (2A, 2B) for HESP-NLCs group, Group 3 (3A, 3B) for NLCs without drug group, Group 4 (4A, 4B) for HESP-pure drug group, Group 5 (5A, 5B) for positive control group, Group 6 (6A, 6B) for negative control group. Abbreviations: HESP—hesperidin; SLNs—solid lipid nanoparticles; NLCs—nanostructured lipid carriers. G—granulation tissue, S—scab, E—epithelial proliferation, *—mild congestion, **—Lymphatic infiltration; K—homogenous keratinized cells; P—The papillary layer; R—The reticular dermis.

Histopathological studies verified the results obtained from the percentage of wound closure, as shown in Figure 16. Since collagen synthesis plays a fundamental role in the healing of wounds, on the 14th, rats in the treatment groups presented variances in microscopic inflammation compared with the rats in the negative control group as displayed in Figure 16-GP6 (6A, 6B), which proved the typical structure of the intact skin layer of rats, as illustrated. The epidermis comprises a stratified squamous keratinized epithelium, with the cornified layer consisting of flat, homogeneous keratinized cells. Positioned beneath the epidermis is the papillary layer of the dermis, which is a thin layer of loose connective tissue. The reticular dermis is below the papillary dermis and includes numerous hair follicles and sebaceous glands.

In contrast, the positive control in Figure 16-GP5 (5A, 5B) showed scab formation at the wound edge, along with lymphatic infiltration and granulation tissue. Epithelial development at the wound site was absent. The outcome of the HESP pure drug, Figure 16-GP4 (4A, 4B), showed scab formation at the wound edge, the presence of leukocytes in granulation tissue, and mild epithelial regrowth caused by hesperidin anti-microbial activity.

The HESP-SLNs Figure 16-GP1 (1A, 1B) results indicated a moderate degree of lymphocyte infiltration, scab formation atop the newly formed granulation tissue, and epithelialization, which specified an incomplete wound-healing process. Nevertheless, the results for NLCs without HESP, Figure 16-GP3 (3A, 3B), displayed moderate congestion within the granulation tissue under the formed scab at the wound site, lymphatic infiltration, and minimal epithelial proliferation. Instead, the findings for HESP-NLCs, Figure 16-GP2 (2A, 2B), revealed minor lymphatic infiltration, a reduction in inflammation compared to the HESP-SLNs in Figure 16-GP1 (1A, 1B), complete epithelial regeneration covered with keratin, and the presence of newly synthesized collagen fibers, indicating complete wound healing.

Accordingly, this study found that the HESP-NLCs (GP2) group showed the most successful wound-healing outcomes. This was evidenced by complete epithelial regeneration, minimal lymphatic infiltration, and increased collagen deposition and vascularization.

This may be attributed to the presence of terpene hydrocarbons terpinen-4-ol, γ-terpinene, and α-terpinene in addition to tertiary alcohols in TTO’s antimicrobial, antioxidant, and anti-inflammatory activity, and it helps in the regeneration of collagen and promotes the formation of microvessels [93]. On the other hand, hesperidin strengthens capillaries, reducing bleeding and infection, and promotes epithelialization, collagen deposition, and cell proliferation, aiding in wound healing [94,95]. These findings indicate the synergistic effect of TTO and hesperidin in both stimulating cell proliferation and migration, offering a novel approach to treating wounds. These results highly correlated with in vivo wound-healing evaluation studies.

#### 2.12.3. Assessment of Serum Inflammatory Biomarkers

**Figure 17:**
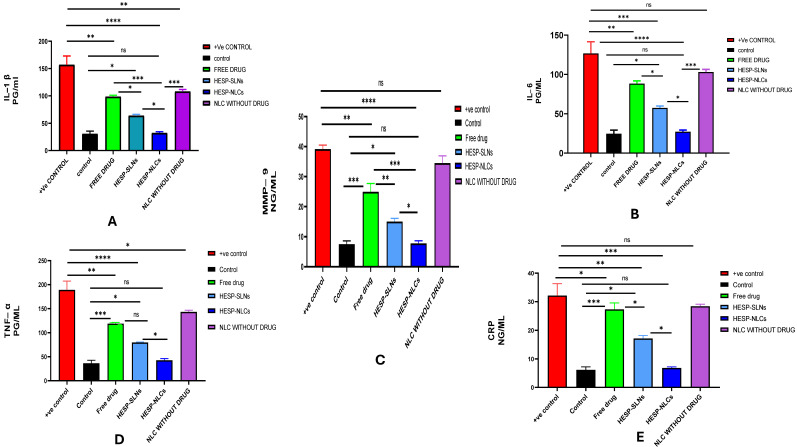
Assessment inflammatory markers, including CRP, IL-6, IL-1, MMP-9, and TNF-α of different animal groups. (**A**) Interleukin-1 beta (IL-1β pg/mL), (**B**) serum interleukin-6 (IL-6 pg/mL), (**C**) matrix metalloproteinase-9 (MMP-9 ng/mL), (**D**) tumor necrosis factor- alpha (TNF-α pg/mL), and (**E**) C-reactive protein (CRP mg/mL). Data are reported as the average ± standard deviation obtained from three independent trials (n = 3). (ns) = not significant; (****) = *p* < 0.0001; (***) *p* < 0.001; (**) = *p* < 0.01; (*) = *p* < 0.1. Abbreviations: HESP—hesperidin; SLNs—solid lipid nanoparticles; NLCs—nanostructured lipid carriers.

The results from inflammatory markers, including CRP, IL-6, IL-1, and MMP-9 in addition to TNF-α, are critical indicators of inflammation and the immune response. As displayed in Figure 17A–E, the outcomes of the investigation reveal that the use of the HESP pure drug (GP4) caused a reduction in all inflammatory biomarkers in contrast to GP5, the positive control (*p* < 0.05), except C-reactive protein (CRP), which displayed no statistically significant change. However, it failed to return normal levels of biomarkers (*p* < 0.001) in contrast to the negative group (GP6). Concerning NLCs without HESP (GP3), the results presented that interleukin-6 (IL-6), matrix metalloproteinase-9 (MMP-9), and CRP did not exhibit significant variation from the positive control (GP5). However, a noticeable difference was observed (*p* < 0.05) in comparison to (GP6) the negative group, as illustrated in Figure 17. Conversely, the incorporation of hesperidin into SLNs (GP1) produced a statistically significant decrease in inflammatory biomarker levels, in contrast to GP5, the positive control (*p* < 0.05), with significant variations, demonstrated when analyzed against each of the negative control (GP6) (*p* < 0.1), the NLCs without HESP(GP3), and the HESP pure drug (GP4) (*p* < 0.05). Hesperidin-loaded NLCs (GP2) also demonstrated a significant reduction in biomarker levels in contrast with the positive control (GP5) (*p* < 0.00001). They were notably different from the NLCs without hesperidin (GP3) and the pure drug (GP4) (*p* < 0.001). Although they revealed a significant variation from the hesperidin-SLNs (GP1) (*p* < 0.05), They did not change significantly from (GP6), the negative control (*p* > 0.1), implying that hesperidin-loaded NLCs effectively restored the normal level of the biomarker.

The HESP-NLCs (GP2) displayed superior evidence and the most remarkable anti-inflammatory influence in returning the inflammatory biomarker levels to normal, such as the control group. These results belong to the possible synergistic anti-inflammatory effect of TTO and hesperidin. Some of the proposed mechanisms of hesperidin are related to free radicals scavenging and pro-inflammatory activity; it also inhibits NF-κB, reducing cytokines that promote inflammation (e.g., IL-1β, IL-8, IL-10, TNF-α, and (MMP-9), increasing the rate of angiogenesis and revascularization, accelerating fibroblast cell proliferation and migration, down-regulating the expression of (MMP-9), and inducing higher re-epithelialization [96,97]. Additionally, terpenes (terpinen-4-ol) can reduce the expression of IL-1, IL-6, and TNF-α, either in specific macrophage cell lines or in vivo models of rats [60].

This result agreed with Mohamed et al. [90], who reported that tea tree oil inhibited the lipopolysaccharide-induced production of (TNF-α), interleukin-1β, and IL-10. It has been shown that terpinen-4-ol suppressed the production of pro-inflammatory cytokines such as tumor necrosis factor (TNF-α) in cultured human monocytes stimulated with lipopolysaccharides.

#### 2.12.4. Investigation of Skin Irritation

**Figure 18:**
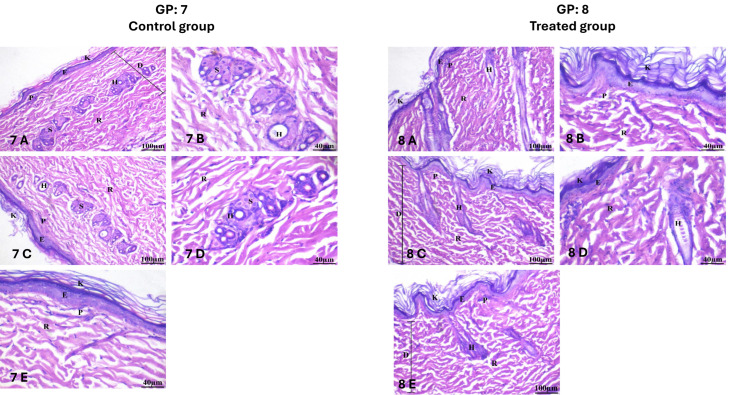
Photomicrographs represent the histopathological differences in intact skin tissues among examined groups; GP7 (7A, 7B, 7C, 7D, and 7E) for Negative Control, GP8 (8A, 8B, 8C, 8D and 8E) for HESP-NLCs-treated group. Abbreviations: HESP—hesperidin; NLCs—nanostructured lipid carriers; E—Epidermis; K—homogenous keratinized cells; P—the papillary layer; D—dermis; R—the reticular dermis; H—hair follicles; S—sebaceous glands.

A skin irritancy experiment was conducted after applying HESP-NLCs topically to the rat’s intact skin for 14 days. The (GP8) rats treated with HESP-NLCs displayed a lack of any cutaneous or inflammatory response such as redness, edema, irritation, swelling, or vascular congestion. No difference was mentioned when compared with the (GP7) negative control. In addition, the histological examination of the (GP7) control group revealed normal skin. The epidermis is covered with a keratin layer. The dermal layer comprises collagen fibers divided into the papillary layer and the reticular layer, which contains hair follicles and the sebaceous gland. Figure 18 GP7 (7A to 7E) shows the histological study of the control group. The epidermis consists of a stratified squamous keratinized epithelium. The cornified layer consists of flat, homogenous keratinized cells. Underneath the epidermis is a film of loosened connective tissue considered the dermal papillary layer. The reticular dermis is situated beneath the papillary dermis. The dermis includes an extensive number of follicles of hair along with sebaceous glands.

In Figure 18 GP8 (8A to 8E), the histological study treated with HESP-NLCs displayed that the epidermis is a stratified squamous keratinized epithelium. Flat, uniform keratinized cells form the cornified layer. Comparable to the histopathological analysis of GP7, the papillary layer of the dermis is a thin film of connective tissue that lies under the epidermis. The reticular dermis lies beneath the papillary. The dermis has many hair follicles and sebaceous glands, which indicate the absence of any prominent inflammation, hyperkeratosis, redness, edema, congestion, or irritation.

Therefore, there were no remarkable variations in (GP8) treatment with HESP-NLCs compared with the (GP7) negative control or untreated. Referring to these results, the proposed TTO-loaded HESP-NLCs are safe and competent for topical application to the skin. This result agreed with what was previously reported by Najafi-Taheret et al. [98], and Vairoet et al. [99]. 

## 3. Materials and Methods

### 3.1. Materials

Hesperidin (HESP purity ≥ 98%) was purchased from Sigma-Aldrich (St. Louis, MO, USA). Stearic acid was obtained by SA; Merck, Darmstadt, Germany, and Compritol^®^ ATO 888 (glyceryl behenate) was provided by Gattefossé, Neuilly-sur-Seine, France. Span^®^ 60 was bought from EL-Nasr Pharmaceutical Chemicals Company, Abu Zaabal, Cairo, Egypt. Pluronic^®^ F127 was procured from Sigma-Aldrich (St. Louis, MO, USA). Tea tree oil and other essential oils, such as lavender, eucalyptus, thyme, clove, and camphor, were acquired by Sigma–Aldrich, Mumbai, India. Potassium hydroxide, Sodium chloride, Sodium dihydrogen phosphate dihydrate, and Potassium chloride were gifted by El-Gomhoria for Chemistry Industrial, Giza, Egypt. Misr University provided Wistar albino rats for the Science and Technology animal center (6th October City, Giza, Egypt). All used solvents and compounds were of analytical grade.

### 3.2. Experimental Design Construction of HESP-SLNs

To determine what factors may have a substantial impact on the SLNs’ attributes, the relevant preliminary research was carried out. The optimization formulation process containing HESP-SLNs was conducted utilizing the 2^4^ factorial design, employing the Design Expert^®^ software (Stat-Ease, Minneapolis, MN, USA) version 13. As shown in Table 7, the investigation focused on four factors (independent factors): lipid type (X1), lipid conc (%) (X2), surfactant type (X3), and sonication amplitude (%) (X4), whereas, the size of particle (nm) (Y1), polydispersibility index (Y2), zeta potential (MV) (Y3), and encapsulation efficiency (%) (Y4) were designated as responses (dependent factors). A statistical study was carried out using (ANOVA) analysis of variance at a 95% level of significance (*p* < 0.05) to estimate the data provided from the responses and the impact of the variable on the preparation of HESP-SLNs.

### 3.3. Preparation of Hesperidin-Loaded Solid Lipid Nanoparticles

HESP-SLNs were produced using a modified high-speed stirring and ultrasonication method, where Stearic acid or Compritol^®^ ATO 888 was utilized as a solid lipid. The clear and homogenous lipid and drug blend was heated at 80 °C to produce the oil phase. For the aqueous phase, 10 mL of deionized water was employed, and a weighed surfactant (Span^®^ 60 or Pluronic^®^ F127) was inserted. At 80 °C, the lipid and aqueous phases were heated at the same temperature. The dropwise aqueous phase was incorporated into the lipid phase and mixed for 10 min with a high-speed magnetic stirrer [100]. Lastly, a pre-emulsion of coarse oil and water was ultrasonically treated for 5 min with a probe sonicator (Ultrasonic processor GE130 with probe CV18, Sonics & Materials Inc., Newtown, CT, USA)for 1 min pulse on, 1 min pulse off, and 20% or 40% amplitude [35,42,101]. Hot dispersion was finally maintained at 4 °C and stored for further investigation [102].

### 3.4. Investigation of HESP-SLNs

#### 3.4.1. Evaluation of PS, ZP, and PDI of HESP-SLNs

The average size of particles (PS), zeta potential (ZP), and polydispersity index (PDI) of the SLNs were established using a dynamic light scattering (DLS) method, and a Zeta sizer instrument (Malvern Instruments, Ltd., Malvern, UK). Deionized water was used to dilute the formulas properly. Triplicate measurements were carried out, and mean and standard deviations were calculated [103].

#### 3.4.2. Percentage of Encapsulation Efficiency (EE%)

First, 1 mL of HESP-SLNs dispersion was ultra-centrifugated (Cooling Centrifuge, Sigma 3K 30, Roedermark, Germany) at 20,000 rpm for an hour at 4 °C to facilitate the sedimentation of the solid lipid. Methanol was utilized to dissolve the residue and then it was examined spectrophotometrically at specific λ_max_ (285 nm) [104], applying UV spectroscopy (Shimadzu-double beam, Kyoto, Japan). The assay was performed three times for each sample, and data were expressed with mean ± SD.

The EE% was computed by applying the following calculation [105].

(1) $$EE\;(\%)=\frac{Amount\;of\;HESP\;entrapped\;in\;SLNs}{Total\;HESP}\times 100$$

### 3.5. Identifying the Optimized HESP-SLNs

The optimized formula was determined using the desirability function. The optimization conditions were established to select the maximum EE%, and ZP (in an absolute value), and the minimum PS, and PD. The trial with the highest desirable function within the specified constraints was chosen as the optimized formula for additional studies [106].

### 3.6. Improvement of Optimized HESP-SLNs Properties Producing Essential Oil-Loaded HESP-NLCs

#### 3.6.1. Screening and Selecting of Essential Oils

##### Diffusion Agar Method

The first evaluation of the antimicrobial effectiveness of essential oils, for instance, lavender, eucalyptus, thyme, and tea tree oil, was performed by applying the Clinical and Laboratory Standards Institute (CLSI) guidelines established in 2021 to evaluate microbial susceptibility with agar diffusion techniques [38]. The microbial strains, *Escherichia coli* ATCC 25922, *Pseudomonas aeruginosa* ATCC 27853, and *Staphylococcus aureus* ATCC 25923, exposed to an incubating duration ranging from 18 to 24 h at a controlled temperature of 37 °C, were employed in the preparation of suspensions, demonstrating a calibrated concentration of 0.5 McFarland (1.5 × 10^8^ CFU/mL). The resulting suspensions were subsequently inoculated into an agar medium. Then, 10 μL of the diluted essential oils was accurately applied in discrete spots. The agar dishes were preserved at typical temperatures to aid the droplets’ adsorption, followed by a 24 h incubation period at 37 °C. The antibacterial efficacy was assessed by observing the formation of a zone of inhibition surrounding the applied spot. As a part of the outcome analysis, the inhibitory zone’s clarity was evaluated, its diameter was measured and reported, and the experiment was conducted in triplicates [107].

##### Evaluation of the Minimum Inhibitory Concentration (MIC)

The minimum inhibitory concentration (MIC) is the minimum concentration required to prevent detectable microbial growth. A lower MIC value correlates with increased antimicrobial efficacy of the respective agent. Consequently, the MIC is a comparative metric for measuring the potency of various antimicrobial agents or distinct microbial strains [108]. The MIC was established in the following experimental stage utilizing the microdilution technique. Separated microorganisms were grown on Mueller–Hinton Agar (MHA) at a steady temperature of 37 °C until they entered the exponential increase phase. The successive dilutions were carried out for the oils in a 96-well plate, with 100 µL of Mueller–Hinton Broth (MHB) in each well of the flat-bottomed 96-well microplates, except the first row, which was designated 200 µL of the oils; the obtained findings were presented as mean ± SD [109].

### 3.7. Preparation of Essential Oil-Loaded HESP-NLCs

HESP-NLCs were produced by employing hot melt emulsion, an ultrasonication procedure, and high-speed stirring [110], where 100 mg of HESP was blended with solid and liquid lipids (selected essential oils). The lipid phase was formed when mixed and heated to 85 °C. The aqueous phase comprised 10 mL of deionized water to which 2% (*w*/*v*) stabilizer was added. Concurrently, for ten minutes each, the aqueous and lipid phases were heated to an identical temperature of 85 °C. Employing a high-speed magnetic stirrer, the aqueous phase was incorporated gradually into the lipid phase and mixed for 5 min [111,112]. The dispersion was exposed to probe sonicator equipment (GE130, probe CV18, USA) for 5 min 1 min pulse on, 1 min pulse off), at 20% amplitude sonication. Finally, 10 mL of the produced dispersion was kept at 4 °C for additional investigation [113].

### 3.8. Investigation of HESP-NLCs

#### 3.8.1. Evaluation of PS, ZP, and PDI of HESP-NLCs

As mentioned in Section 3.4.1, the average size of particles (PS), zeta potential (ZP), and polydispersity index (PDI) of the formulated HESP-NLCs were assessed.

#### 3.8.2. Percentage Encapsulation Efficiency (EE%)

The percentage encapsulation efficiency (EE%) was specified by centrifugation, as formerly stated in Section 3.4.2.

### 3.9. Transmission Electron Microscopy (TEM) of Optimized HESP-SLNs and HESP-NLCs

The investigation of the morphology of optimized HESP-SLNs and HESP-NLCs was conducted extensively using Transmission Electron Microscopy (TEM) with a specific mode (JEM-1230, Joel, Tokyo, Japan) [114]. After the samples were placed on a carbon-coated grid, they were dyed using a 1% aqueous solution of phosphotungstic acid (PTA). Before visualization, the samples were left to dry at room temperature [21].

### 3.10. Compatibility Evaluation Optimized HESP-SLNs and HESP-NLCs

#### 3.10.1. Differential Scanning Calorimetry (DSC)

The thermodynamic assessment was conducted to confirm the probable interaction among HESP (pure drug) and individual components of both the optimized HESP-SLNs and HESP-NLCs, utilizing DSC (Shimadzu Corp., Kyoto, Japan) in the 25 to 400 °C temperature range at a consistent heat rate of 10 °C per minute [15,115].

#### 3.10.2. Raman Spectroscopy

The prospective interactions between HESP (pure drug) and additional excipients in each optimized HESP-SLN and HESP-NLC were evaluated independently using the Raman spectrometer [116]. Raman spectrum was recorded utilizing a Horiba lab RAM HR evolving visible single spectrometer in (Horiba, Edison, NJ, USA) the 532 nm He-Cd edge laser line with diffraction 1800 (450–850 nm), ND filter 0.01%, an acquisition period of 20 s, aggregation with no lag duration, spike filter, and X0_VIS_LWD objective. Evaluation procedures were conducted at ambient temperature with wavelengths ranging from 100 to 3500 cm^−1^ [117]. Any alterations, peak shifts, or broadening were precisely recorded.

### 3.11. In Vitro Release Study of Optimized HESP-SLNs and HESP-NLCs

The in vitro release profile of optimized HESP-SLNs and HESP-NLCs was determined using the dissolution apparatus II model. In the first step, the dialysis membrane (12,000–14,000 Mwt cutoff) was immersed at room temperature in a phosphate-buffered saline solution (PBS) with a pH of 6.8 for the entire night. After that, a plastic cylinder tube was filled with 1 mL of the formula equal to 10 mg of HESP. A dialysis membrane was appropriately wrapped around a single end of the tube, and the other end was attached to the shaft of the dissolution equipment. At 37 °C, 100 mL of PBS (with a pH of 6.8) was used as the dissolution medium, and the apparatus vessel was constantly stirred at 100 rpm through a paddle [118]. At an appropriate interval, 1 mL of release media was removed and substituted with an equivalent volume of fresh media. Samples were withdrawn at 1, 2, 3, 4, 5, and 6 h, and the concentration of HESP was detected at λ_max_ 285 nm. Three runs of the experiment were carried out. The percentage of total drug release was estimated in correlation with the initial quantity of incorporated HESP. The release kinetics was established to investigate the release mechanisms, and several mathematical models, including zero-order, first-order, Higuchi, and Korsmeyer–Peppas models, were fitted to the release data. A regression coefficient (R^2^) was determined to establish the model that most accurately fit the data [53].

### 3.12. Effect of Storage Conditions on the Optimized HESP-SLNs and HESP-NLCs

The optimized HESP-SLNs and HSEP-NLCs were stored for three months at 25 °C and 4 °C, respectively. The withdrawn samples were evaluated in terms of PS, PDI, ZP, and EE%. In addition, a visual inspection was performed to detect any sedimentation or indications of particle aggregation. The results obtained were statistically assessed using *t*-test analysis. To ensure accuracy, the trials were conducted three times and represented as mean ± SD [119].

### 3.13. Determination of In Vitro Antibacterial Properties

The antibacterial effectiveness of HESP (pure drug), HESP-SLNs, HESP-NLCs, and NLCs without HESP was examined using the agar diffusion technique. To create a homogeneous microbial growth plate, the different bacterial strains were placed in a broth medium at 37 °C for 24 h. The subsequent procedure involved the dissemination of entire night cultures on the agar dishes. To determine antibacterial efficacy, the bacteria types utilized were *Pseudomonas aeruginosa* KCTC 2513 (Gram-negative) and *Staphylococcus aureus* ATCC 25923 (Gram-positive). Ultimately, the incubator encompassed petri dishes at a consistent 37 °C for an additional full day. To determine the antibacterial effects, the diameter of the inhibitory zone was measured; this experiment was conducted in triplicate [103].

### 3.14. Cytotoxicity Assay

To evaluate the cytotoxicity effect, Nawah Scientific Inc. (Cairo, Egypt) supplied an HSF (human skin fibroblast) cell line. Cells were cultured at 37 °C in Dulbecco’s Modified Eagle Medium (DMEM) moistened 5% (*v*/*v*) CO_2_, treated using 100 mg per ml streptomycin and 100 units per ml penicillin, along with 10% heat-inactivated fetal bovine serum (FBS). The viability of the cell was estimated through the sulforhodamine B (SRB) colorimetric assay. Then, 96-well plates were loaded with 100 μL suspension of cell samples (5 × 10^3^ cells), and the cells were kept in media for 24 h. Cells were subjected to 100 μL of media, including the optimized HESP-NLCs formula, at different strengths (0.1, 1, 10, 100 μg/mL). After 72 h of drug exposure, the cells were then fixed by incorporating the medium with 150 μL of 10% trichloroacetic acid (TCA), allowing them to incubate at 4 °C for one hour. After eliminating the TCA solution, deionized water was used to rinse the cells five times. Following adding 70 μL containing SRB (0.4% *w*/*v*) solution, the mixture was kept in a dark place at ambient temperature for ten minutes. The plates were rinsed with 1% acetic acid three times and then allowed to dry for the entire night. Finally, the protein-bound SRB stain was liquified with 150 μL containing tris aminomethane base solution (TRIS) (10 mM), and the absorbance at 540 nm was assessed through a BMG LABTECH FLUOstar Omega microplate reader. The relative cell viability and the cytotoxic impact of the experiments on the cell culture were then determined using the obtained absorbance values (BMG LABTECH, Ortenberg, Germany) [120].

### 3.15. In Vivo Study

#### 3.15.1. Experimental Animals

For the in vivo examinations, thirty-eight healthy normal albino Wistar rats weighing 200 ± 20 g each were included in the experiment, where GP1, GP2, GP3, GP4, GP5, and GP6 were (n = 5), and GP7 and GP8 were (n = 4). The investigation was carried out adhering to the National Institute of Health’s recommendations for the use and care of animal experiments (NIH Publication NO. 8023, changed 1978) and the Animal Research Reporting of In Vivo Experiments (ARRIVE) protocol. Furthermore, the research study was approved by the Research Ethics Committee of Cairo University’s Faculty of Pharmacy with number (PI 2945) on the date of (28 March 2022). An animal house, the pharmacy faculty, Cairo University, Egypt, provided the rats. All animals with underlying health issues or abnormal behaviors were excluded. After an adaptation period of 72 h, the experiments began. Animals were housed in clean, dry cages with adequate space for safe movement in a comfortable environment maintained at 21 °C and 50–55% humidity with a 12 h light/dark cycle. The animals had easy access to food and water, and the cages were equipped with satisfactory ventilation, proper illumination, and appropriate sanitation [121]. As shown in Table 8, thirty rats were randomly allocated into six groups; each group had five rats (n = 5). The study design was parallel design open-label.

#### 3.15.2. Wound Excision Induction

The Wistar albino rats of the subsequent groupings (GP1, GP2, GP3, GP4, and GP5) were trimmed on the dorsal side and were sanitized efficiently using a 70% ethanol solution. Following the disinfection process, the full-thickness incision of 2 cm on the dorsal side was made under anesthesia using an intraperitoneal ketamine/xylazine blend (0.1 mL per kg weight of rat, consisting of 100 mg per kg of ketamine and 10 mg per kg of xylazine) in a 1:1 ratio [122]. The formulae were installed topically on the inducted wound twice daily for 14 days.

#### 3.15.3. Assessment of the Percentage of Wound Closure

Collectively, GP1, GP2, GP3, GP4, and GP5 were treated two times a day throughout 14 days. On days 7 and 14, the subsequent equation was used to determine the percent of wound closure. The data were presented as means and standard deviations. The test of significance was assessed by analyzing variance (ANOVA) (*p* < 0.05), via GraphPad version 3.1 (InStat, GraphPad Software, Boston, MA, USA).

(2) $$Wound\;closure\;\%=\frac{{Wt}_{0}-{Wt}_{i}}{{Wt}_{0}}\times 100$$

where Wt_0_ is the primary wound area, and Wt_i_ is the determined wound area at a specified time.

#### 3.15.4. Histopathology Study

On the 14th day of the experiment, a histopathological investigation was carried out. Tissue samples of all groups were collected. The skin tissue of each group was excised and preserved in 10% formalin within a phosphate buffer solution. This was followed by embedding in paraffin blocks. Subsequently, the tissues that had undergone processing were cut into sections with a microtome, each measuring 3–5 µm in thickness, and then subjected to staining using hematoxylin and eosin. Microscopic evaluation was done blindly using a light microscope [123].

#### 3.15.5. Assessment of Serum Inflammatory Biomarkers

Following the termination of the experiment (day 14), the collected blood samples from three anesthetized rats from each group to evaluate interleukin-6 (IL-6), high-sensitivity C-reactive protein (hs-CRP), interleukin-9 (IL-9), matrix metalloproteinase-9 (MMP- 9), and tumor necrosis factor alpha (TNF-α) levels utilizing (ELISA) enzyme-linked immunosorbent assay, following the instructions stated by the manufacturer [124]. For each set of data, means and standard deviations (SDs) are displayed. The statistical program GraphPad version 3.1 (InStat, GraphPad Software, Boston, MA, USA) was deployed to compute the analysis of variance (ANOVA) statistically (*p* < 0.05).

#### 3.15.6. Investigation of Skin Irritation

An experiment was conducted to assess the possibility of skin irritation of the essential oil-loaded HESP-NLCs on eight Wistar rats (200 ± 20 g) divided equally into two groups (n = 4). As shown in Table 8, eight rats were randomly allocated into two groups; each group had four rats (n = 4). The study design was parallel design open-label. The formula was placed daily on the dorsal side of the intact skin of the rat for 14 days. Where GP7 is negative control that received distilled water, and GP8 received HESP-NLCs. Evidence of any edema, erythema, or cutaneous reactions was monitored. Immediately following the experiment, rat skin specimens were taken for additional histological analysis [125,126].

### 3.16. Statistical Analysis

The findings were displayed as the average with standard deviation. The statistical evaluation utilized the statistical program GraphPad version 3.1, InStat, GraphPad Software, Boston, MA, USA. Statistical assessments were computed using the Tukey post hoc analysis following a one-way analysis of variance (ANOVA) (*p* < 0.05).

## 4. Conclusions

The HESP-SLNs were effectively developed in this study, and the optimized formula was selected utilizing Design Expert, which was further improved by incorporating TTO into HESP-NLCs. Notably, the improved formula enhanced the encapsulation capacity, solubility, and stability of both HESP and TTO. It also showed synergistic therapeutic efficacy, resulting in the improvement of in vitro antimicrobial activity and anti-inflammatory action. Furthermore, either a cytotoxicity assay or skin irritation test confirmed the safety behavior of TTO-incorporated HESP-NLCs for topical application on the skin; specifically, in vivo studies proved the HESP-NLCs were highly effective in promoting faster epithelialization and improved wound closure; thus, the finding indicated the TTO-loaded HESP-NLCs are a potential method for effectively and safely treating wounds. We recommend conducting further clinical studies to explore the potential therapeutic applications of TTO-loaded hesperidin-NLCs, consolidating our research finding.

## Figures and Tables

**Table 1:** Experimental runs, independent variables, and the response of the 2^4^ factorial design of HESP-SLNs.

No.	(X1) Lipid Type	(X2) Lipid Concentration	(X3) Surfactant Type	(X4) Sonication Amplitude (%)	(Y1) PS (nm)	(Y2) ZP (mV)	(Y3) PDI	(Y4) EE (%)
HESP-SLNs1	Stearic acid	2%	Span^®^ 60	40%	275 ± 2.26	−31.7 ± 2.47	0.339 ± 0.132	53.6 ± 2.90
HESP-SLNs2	Stearic acid	2%	Span^®^ 60	20%	252 ± 3.12	−36.2 ± 1.52	0.246 ± 0.155	55.2 ± 3.49
HESP-SLNs3	Compritol^®^ ATO 888	2%	Span^®^ 60	40%	300.8 ± 5.23	−23.5 ± 4.25	0.366 ± 0.191	59.2 ± 2.14
HESP-SLNs4	Compritol^®^ ATO 888	2%	Span^®^ 60	20%	267.7 ± 1.89	−35.4 ± 3.53	0.317 ± 0.129	65.8 ± 3.02
HESP-SLNs5	Stearic acid	2%	Pluronic^®^ F127	40%	363.9 ± 3.14	−25.3 ± 2.59	0.476 ± 0.151	51.8 ± 0.86
HESP-SLNs6	Stearic acid	2%	Pluronic^®^ F127	20%	354.4 ± 2.33	−22.9 ± 2.12	0.435 ± 0.129	52.4 ± 2.13
HESP-SLNs7	Compritol^®^ ATO 888	2%	Pluronic^®^ F127	40%	375.4 ± 2.82	−23.3 ± 0.52	0.571 ± 0.161	58.6 ± 1.13
HESP-SLNs8	Compritol^®^ ATO 888	2%	Pluronic^®^ F127	20%	369.5 ± 1.42	−32.3 ± 3.71	0.492 ± 0.146	62.4 ± 1.27
HESP-SLNs9	Stearic acid	5%	Span^®^ 60	40%	302.7 ± 1.75	−33.3 ± 3.85	0.294 ± 0.132	72.4 ± 1.7
HESP-SLNs10	Stearic acid	5%	Span^®^ 60	20%	280 ± 1.35	−39.4 ± 0.92	0.239 ± 0.012	88.2 ± 2.09
HESP-SLNs11	Compritol^®^ ATO 888	5%	Span^®^ 60	40%	354.1 ± 2.81	−30.4 ± 2.45	0.391 ± 0.131	79.3 ± 2.87
HESP-SLNs12	Compritol^®^ ATO 888	5%	Span^®^ 60	20%	284 ± 2.07	−36.9 ± 1.03	0.248 ± 0.091	85.1 ± 2.12
HESP-SLNs13	Stearic acid	5%	Pluronic^®^ F127	40%	383.5 ± 1.26	−29.4 ± 3.79	0.334 ± 0.174	55.8 ± 1.36
HESP-SLNs14	Stearic acid	5%	Pluronic^®^ F127	20%	370 ± 2.58	−37.1 ± 2.19	0.311 ± 0.231	60.4 ± 1.10
HESP-SLNs15	Compritol^®^ ATO 888	5%	Pluronic^®^ F127	40%	495 ± 1.70	−24.4 ± 1.74	0.450 ± 0.197	64.2 ± 1.34
HESP-SLNs16	Compritol^®^ ATO 888	5%	Pluronic^®^ F127	20%	380.3 ± 2.96	−34.8 ± 0.93	0.378 ± 0.143	66.3 ± 2.67

Note: Data are reported as the average ± standard deviation obtained from three independent trials (n = 3). Abbreviations: PS—particle size; PDI—polydispersity index; ZP—zeta potential; and EE (%)—entrapment efficiency percentage; HESP—hesperidin; SLNs—solid lipid nanoparticles.

**Table 2:** Output data of the 2^4^ factorial design analysis of HESP-SLNs.

Response	Y1: PS (nm)	Y2: ZP (Absolutes)	Y3: PDI	Y4: EE (%)
*p*-value	*p* < 0.0001	*p* < 0.0001	*p* < 0.0001	*p* < 0.0001
R^2^	0.9586	0.9091	0.9803	0.9911
Adj. R-squared	0.9388	0.8659	0.9708	0.9869
Pre. R-squared	0.9038	0.7890	0.9541	0.9794
Adequate precision	23.539	16.5419	35.5317	49.5395
Significant factors of the optimized (HESP-SLNs 10)	X1, X2, X3, X4	X1, X2, X3, X4	X1, X2, X3, X4	X1, X2, X3, X4
The predicted value of the optimized (HESP-SLNs 10)	277.97	40.8	0.229	81.8
The observed value of the optimized (HESP-SLNs 10)	280	39.4	0.239	88.2

Abbreviations: PS—particle size; PDI—polydispersity index; ZP—zeta potential; and EE (%)—entrapment efficiency percentage; HESP—hesperidin; SLNs—solid lipid nanoparticles.

**Table 3:** Diameter of inhibition zone of essential oils (mm).

Essential Oils/ Microbial Strains	Diameter of Inhibition Zone (mm)		
	*Escherichia coli* ATCC 25922	*Pseudomonas aeruginosa* ATCC 27853	*Staphylococcus aureus* ATCC 25923
Lavender Oil	15 ± 1.5	22 ± 2.5	30 ± 3
Eucalyptus Oil	15 ± 1	11 ± 2	6 ± 1
Thyme Oil	30 ± 3	27 ± 4	29 ± 3
Tea Tree Oil	35 ± 2.5	37 ± 2	45 ± 2

Note: data are reported as the average ± standard deviation obtained from three independent trials (n = 3).

**Table 4:** Minimum inhibitory concentration (MIC); values of essential oils against bacteria (mg/mL).

Essential Oils/ Microbial Strains	Minimum Inhibitory Concentration (mg/mL)		
	*Escherichia coli* ATCC 25922	*Pseudomonas aeruginosa* ATCC 27853	*Staphylococcus aureus* ATCC 25923
Lavender Oil	6.1 ± 0.3	6.3 ± 0.2	2.1 ± 0.7
Eucalyptus Oil	3.3 ± 0.4	2.7 ± 0.2	3.1 ± 0.5
Thyme Oil	1.9 ± 0.4	2.2 ± 0.3	2.3 ± 0.2
Tea Tree Oil	1.6 ± 0.1	1.2 ± 0.2	1.2 ± 0.3

Note: data are reported as the average ± standard deviation obtained from three independent trials (n = 3).

**Table 5:** Impact of storage conditions on the optimized HESP-SLNs.

**Parameter**	**Freshly Prepared**	**HESP-SLNs After 3 Months of Storage at 4 °C**	**HESP-SLNs After 3 Months of Storage at 25 °C**
PS (nm)	280 ± 1.35	295 ± 1.74	290 ± 2.13
ZP (mV)	−39.4 ± 0.92	−37.23 ± 0.24	−35.01 ± 1.32
PDI	0.239 ± 0.012	0.247 ± 0.56	0.251 ± 0.14
EE (%)	88.2 ± 2.09	85.2 ± 1.36	86.72 ± 1.58

Notes: Data are reported as the average ± standard deviation obtained from three independent trials (n = 3). Abbreviations: PS—particle size; PDI—polydispersity index; ZP—zeta potential; and EE%—entrapment efficiency percentage; HESP—hesperidin; SLNs—solid lipid nanoparticles.

**Table 6:** Impact of storage conditions on HESP-NLCs.

Parameter	Freshly Prepared	HESP-NLCs After 3 Months of Storage at 4 °C	HESP-NLCs After 3 Months of Storage at 25 °C
PS (nm)	300 ± 5.21	315.93 ± 1.84	309.5 ± 2.01
ZP (mV)	−39.4 ± 0.42	−38.29 ± 0.62	−37.21 ± 1.63
PDI	0.272 ± 0.023	0.279 ± 0.03	0.302 ± 0.15
EE (%)	93.7 ± 1.55	88.57± 2.36	91.37 ± 1.47

Notes: Data are reported as the average ± standard deviation obtained from three independent trials (n = 3). Abbreviations: PS—particle size; PDI—polydispersity index; ZP—zeta potential; and EE%—entrapment efficiency percentage; HESP—hesperidin; NLCs—nanostructured liquid carriers.

**Table 7:** Full factorial design for the optimization of HESP-SLNs formulae.

**Factors (Independent Variable)**	**Low Levels**	**High Levels**
X1: Lipid type	Compritol^®^ ATO 888	Stearic acid
X2: Lipid conc	2%	5%
X3: Surfactant type	Pluronic^®^ F127	Span^®^ 60
X4: Sonication amplitude	20%	40%
**Responses (Dependent Variables)**	**Desirability Constraints**	
Y1: PS (nm)	Minimize	
Y2: ZP (absolute values) (mv)	Maximize	
Y3: PDI	Minimize	
Y4: EE (%)	Maximize	

Abbreviations: PS: particle size, ZP: zeta potential, PDI: polydispersibility index, EE%: entrapment efficiency percentage, HESP: hesperidin, SLNs: solid lipid nanoparticles.

**Table 8:** In vivo wound-healing assay groups.

Group Name	n	Treatment	Experiment
GP1	5	HESP-SLNs	Wound-healing assay groups
GP2	5	HESP-NLCs	
GP3	5	NLCs without HESP	
GP4	5	HESP pure drug	
GP5	5	Positive Control (untreated group)	
GP6	5	Negative Control (Distilled water)	
GP7	4	Negative Control (Distilled water)	Investigation of skin irritation groups
GP8	4	HESP-NLCs	

Abbreviations: HESP—hesperidin; SLNs—solid lipid nanoparticles, NLCs—nanostructured lipid carriers.

## Data Availability

The data are confidential.
